# The Use of Various Types of Waste Paper for the Removal of Anionic and Cationic Dyes from Aqueous Solutions

**DOI:** 10.3390/molecules29122809

**Published:** 2024-06-12

**Authors:** Tomasz Jóźwiak, Urszula Filipkowska, Anna Bednarowicz, Dorota Zielińska, Maria Wiśniewska-Wrona

**Affiliations:** 1 Department of Environmental Engineering, University of Warmia and Mazury in Olsztyn, Warszawska St. 117a, 10-957 Olsztyn, Poland; urszula.filipkowska@uwm.edu.pl; 2 Lukasiewicz Research Network—Lodz Institute of Technology, 19/27 M. Sklodowskiej-Curie Str., 90-570 Lodz, Poland; anna.bednarowicz@lit.lukasiewicz.gov.pl (A.B.); dorota.zielinska@lit.lukasiewicz.gov.pl (D.Z.); maria.wisniewska-wrona@lit.lukasiewicz.gov.pl (M.W.-W.); 3 Textile Institute, Lodz University of Technology, 116 Żeromskiego Street, 90-924 Lodz, Poland

**Keywords:** waste paper, newsprint, LWC, office paper, corrugated cardboard, unconventional sorbent, sorption, anionic dyes, cationic dyes

## Abstract

This study examined the possibility of using various types of waste paper—used newsprint (NP), used lightweight coated paper (LWC), used office paper (OP), and used corrugated cardboard (CC)—for the removal of anionic dyes, Acid Red 18 (AR18) and Acid Yellow 23 (AY23), and cationic dyes, Basic Violet 10 (BV10) and Basic Red 46 (BR46), from aqueous solutions. The scope of this research included the characterization of sorbents (FTIR, SEM, BET surface area, porosity, pH_PZC_, effectiveness of water coloration), determination of pH effect on the effectiveness of dye sorption, sorption kinetics (pseudo-first-order model, second-order model, intraparticular diffusion model), and the maximum sorption capacity (Langmuir models and Freundlich model) of the tested sorbents. The use of waste paper materials as sorbents was found to not pose any severe risk of aquatic environment contamination. AR18, AY23, and BV10 sorption intensities were the highest at pH 2, and that of RB46 at pH 6. The waste paper sorbents proved particularly effective in removing cationic dyes, like in the case of, e.g., NP, which had a sorption capacity that reached 38.87 mg/g and 90.82 mg/g towards BV10 and BR46, respectively, and were comparable with that of selected activated carbons (literature data).

## 1. Introduction

Dyes are substances that selectively absorb electromagnetic radiation in the visible range (from 380 nm to 780 nm). Most often, they are organic compounds with a complex chemical structure, the color of which depends on chromophore type [1]. They are used in industry to impart color on various materials, with their largest demand observed in the textile, tanning, and paper industries [2]. As dyeing technologies are sometimes imperfect, a vast portion of dyes may end up in post-production water [3] and, further, may contaminate the natural environment due to ineffective methods for wastewater decolorization.

Dyes contained in industrial wastewater may pose a severe threat to the natural environment. Colored substances are particularly harmful to aquatic ecosystems [4]. Even their small concentrations in natural waters are very visible and disturb the aesthetics of the landscape. However, a far more serious consequent problem is that they hinder sunlight availability to autotrophs, which results in the inhibition of the primary production [5]. In addition, certain dyes react directly with oxygen dissolved in water, which, combined with inefficient photosynthesis, poses the risk of development of anaerobic conditions [6]. Certain dyes may also be directly toxic to aquatic organisms [7]. As a result, contamination of water reservoirs with dyes may lead to diminished biodiversity and even the complete collapse of aquatic ecosystems.

The potential consequences of environment pollution from dyes have prompted use of the most effective methods for colored wastewater treatment. Today, sorption is believed to be one of the most effective and environmentally friendly methods of wastewater decolorization [8]. It involves binding contaminants on a sorbent’s surface. The most popular group of sorbents is activated carbons [9], which prove highly effective in binding most dyes. The high sorption capacity of activated carbons results from their very large specific surface area, usually over 500 m^2^/g [10]. However, the high costs of their production and regeneration are their obvious disadvantage [11]. For this reason, their cheaper substitutes are currently being sought.

Raw materials to be used for sorbent production are expected to be both inexpensive and widely available. In recent years, great hopes have been fostered in the application of waste materials from the agri-food industry as unconventional sorbents [12]. The largest group of sorption materials tested so far was lignocellulosic plant biomass [13,14], including stems [15] and leaves of crops [16,17], seed hulls [18,19], nut shells [20,21], as well as fruit [22,23] and vegetable peels [24,25]. Ample studies have also been carried out on the removal of dyes using waste from the wood industry, such as sawdust [26,27], bark [28,29], cones [30,31], or tree leaves [32,33]. In some cases, scientists have reported that the sorption capacities of the tested plant sorbents were similar to those of some types of activated carbons [34,35]. The sorption capacity of plant biomass is owed mainly to its polysaccharides (cellulose, hemicellulose) and lignin. The disadvantage of using waste plant biomass as a sorbent is usually its high susceptibility to rotting, which hampers its storage and application.

An alternative type of high-cellulose waste material is waste paper, which is generally more stable than plant waste. It is defined as unnecessary or damaged paper products that can be reused for purposes other than those originally intended [36]. The global consumption of paper products is over 414 million tons, of which approximately 60% is recycled (2022). For this reason, waste paper is perceived as a widely available and cheap material. It is most often used as one of the raw materials for new paper production [37]. However, its high content of polysaccharides indicates its potential usability as a viable sorbent.

The sorption capacity of waste paper towards dyes may theoretically depend on the type of paper (ratios of polysaccharides and lignin), its grammage, porosity, as well as the content and chemical composition of fillers and coatings. The following types of waste paper can be distinguished based on paper type:

Used newsprint (NP)—which is a material that can be sourced from outdated newspapers (letterpress prints), old receipts, or tickets. It usually has a grammage of 50 g/m^2^. It is one of the cheapest types of paper produced mainly from wood pulp of coniferous trees (mechanical pulp), which, due to cost constraints, is not subject to delignification. Due to the high lignin content (up to 30%), this paper darkens under the influence of sunlight and also becomes brittle. It often does not contain any glues or fillers, which makes it very absorbent [38,39]. Newsprint contains certain amounts of printing ink made of pigments, resins and auxiliary substances. Its global production is approximately 22 million tons per year (2022) [40].

Used lightweight coated paper (LWC), used gloss paper—which is wood-based paper that can be sourced from illustrated magazines, advertising brochures, or color catalogs (generally offset printing). Its grammage is typically 35–80 g/m^2^. It is made of a mixture of wood pulp (mechanical pulp) and cellulose bulk, covered with a mineral coating (e.g., kaolin, talc or calcium carbonate) or a polymer coating (e.g., polyethylene or silicone) [41]. The global production of this paper reaches 21 million tons per year (2023).

Used office paper (OP)—which is wood-free paper used mainly for printing/photocopying documents. Its grammage is typically 80 g/m^2^. It is mainly produced from bleached cellulose pulp. Due to its low lignin content (<5%), this paper material is very robust. The spaces between the cellulose fibers are filled with calcium carbonate or kaolin. The annual global production of office paper is approximately 83 million tons (2023) [42].

Used corrugated cardboard (CC)—which is a type of multi-layer (usually 3-layer) paper material, commonly found in various types of packaging/boxes. Its grammage is >315 g/m^2^. It is usually produced from two types of paper layers: liners (kraftliners), which are stiff, straight cardboard sheets, and flutings (testliners), which are a thinner, wavy layer of flexible paper, usually located between two liner layers. The liners are most often produced from unbleached kraft pulp (primary pulp produced in the Kraft process), often with a small admixture of virgin wood pulp. The structure of the cellulose fibers of the liners is strongly interwoven, which significantly increases the mechanical strength of the layer. The flutings (testliners) are usually produced entirely from recycled cellulose fibers, the quality of which is lower than that of virgin fibers. However, the use of virgin wood pulp in the production of testliners is not excluded. For this reason, the testliners have lower strength than the liners [43]. Testliners and liners are usually joined together using small amounts of starch glue. Calcium carbonate often serves as a filler in corrugated cardboard. The estimated annual production of corrugated cardboard is approximately 53 million tons (2022) [44].

The content of dyes/pigments in the mass of waste paper may be up to 2% [45]. Therefore, the use of waste paper as a sorbent poses a certain risk of secondary contamination of the treated solutions with colored substances.

In view of the above data, the present study examined the possibility of using the following types of waste paper, NP, LWC, OP, and CC, as unconventional sorbents for removing anionic dyes, Acid Red 18 (AR18) and Acid Yellow 23 (AY23), and cationic dyes, Basic Violet 10 (BV10) and Basic Red 46 (BR46), from aqueous solutions. Given the pursuit of maximal cost reduction related to sorption materials, the waste paper analyzed in this study was not purified or modified in any way. The scope of research included characterization of sorbents (FTIR, SEM, BET analysis, porosity, pH_PZC_), determination of solution pH on the effectiveness of dye sorption, sorption kinetics, and the maximum sorption capacity of the tested sorbents. Due to the presence of colored substances in some types of waste paper, preliminary research was first carried out to determine the effectiveness of dyeing aqueous solutions with the tested sorbents.

## 2. Results and Discussion

### 2.1. Characterization of the Tested Sorbents (FTIR Analysis, Specific Surface Area, SEM Analysis)

#### 2.1.1. FTIR Analysis

**Figure 1:**
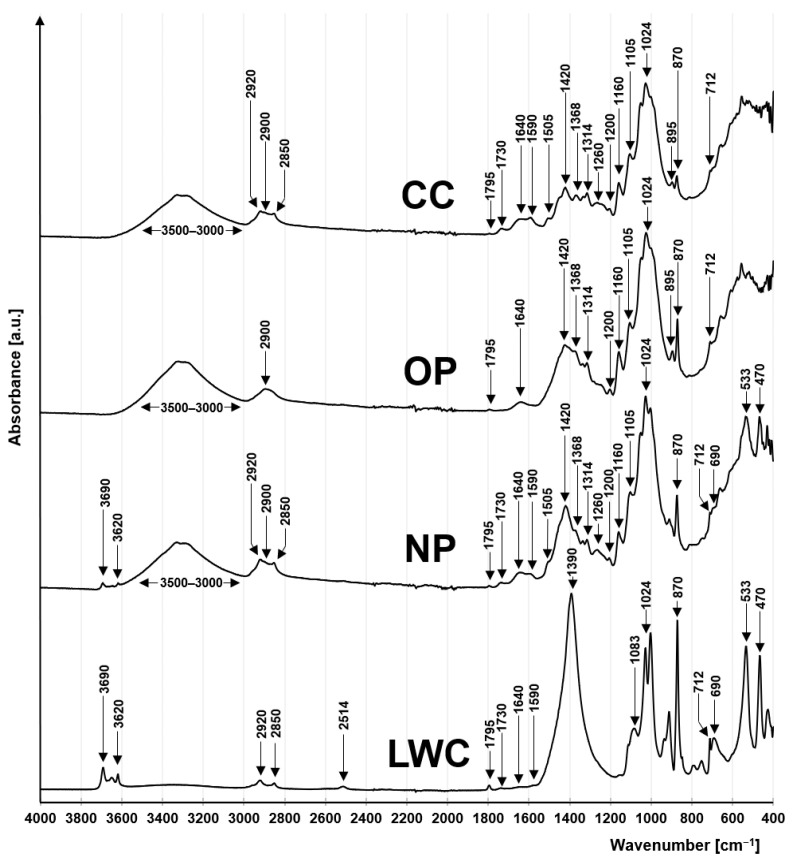
FTIR spectra of NP, LWC, OP, and CC.

The FTIR spectra of the analyzed waste paper sorbents (NP, LWC, OP, and CC) were typical of the high-cellulose materials (Figure 1). Peaks visible at 1200 cm^−1^; 1160 cm^−1^; 1105 cm^−1^; and 1024 cm^−1^ were ascribed to the glycosidic C-O-C bond between the aromatic rings of cellulose and hemicellulose [46,47,48,49]. A small peak visible at 895 cm^−1^ in OP and CC spectra indicated the stretching of saccharide rings [50]. Other characteristics of the holocelluloses were peaks visible on the NP, OP, and CC spectra at 2900 cm^−1^ and 1420 cm^−1^, which correspond to the stretching vibrations of -CH_2_, [51], and also peaks at 1368 cm^−1^ and 1314 cm^−1^, corresponding to the bending and wagging vibrations of -CH_2_ [46,52,53].

The NP and CC spectra also showed peaks pointing to the presence of lignin in the material, i.e., peaks visible at 1505 cm^−1^ and 1260 cm^−1^, which were ascribed to the unsaturated C=C bonds of the guaiacol rings (phenylpropane rings) of lignin [46]. The presence of lignin in the sorbents was also indicated by peaks visible at 1730 cm^−1^ and 1590 cm^−1^ and pointed to the presence of carbonyl C=O bonds. Peaks at 2920 cm^−1^ and 2850 cm^−1^ are also typical of lignin and correspond to the stretching of the C-H bond in aromatic methoxyl groups as well as methyl and methylene side chains [54].

The OP spectrum showed no lignin-specific peaks (2920 cm^−1^; 2850 cm^−1^; 1590 cm^−1^; 1505 cm^−1^; and 1260 cm^−1^) due to a very minor lignin content in this material (<1%).

A wide absorption band at 3500–3000 cm^−1^ was ascribed to the stretching of the O-H bond, typical for hydroxyl functional groups, occurring in both saccharides and lignin. In turn, a peak visible at 1640 cm^−1^ corresponded to vibrations of the O-H bond of water contained in the material [49,55].

Clear peaks visible at 870 cm^−1^ and 712 cm^−1^ in the spectra of each sorbent indicate the asymmetric and symmetric bending of the C-O bond of carbonates. They were related to the presence of calcite (calcium carbonate), which is used as a filler in paper products [56,57]. Its presence in the analyzed materials was also indicated by peaks at 2514 cm^−1^ and 1795 cm^−1^, which were particularly noticeable in the LWC spectrum and corresponded to the asymmetric and symmetric vibrations of the C-O bond of carbonates [57]. Due to the high calcite content of the LWC, its spectrum also revealed distinct peaks at 1390 cm^−1^ and 1083 cm^−1^, corresponding to the asymmetric stretching and bending of the C-O bond of the carbonate [56]. The peak visible at 1390 cm^−1^ in the LWC spectrum overlapped with peaks in the range of 1500–1200 cm^−1^, thus making the identification of certain structures that are typical of holocelluloses impossible. In the other spectra (NP, OP, and CC), the peaks characteristic for cellulose and hemicellulose (1420 cm^−1^ and 1105 cm^−1^) overlapped, with some peaks indicating the presence of carbonates in the tested material (1390 cm^−1^ and 1083 cm^−1^).

The NP and LWC spectra show additional peaks, ascribed to the presence of kaolin in the material, as confirmed by bands 3690 cm^−1^ and 3620 cm^−1^, which are characteristic for this aluminosilicate and correspond to O-H and Al-OH bonds. Like calcite, kaolin is a popular filler used in paper materials. Its presence in the NP and LWC was also indicated by peaks visible at 754 cm^−1^, 533 cm^−1^, and 470 cm^−1^, corresponding to the stretching of the Al-O-Si bond, and also a peak at 690 cm^−1^, corresponding to Si-O bond vibration [55].

#### 2.1.2. SEM Analysis

**Figure 2:**
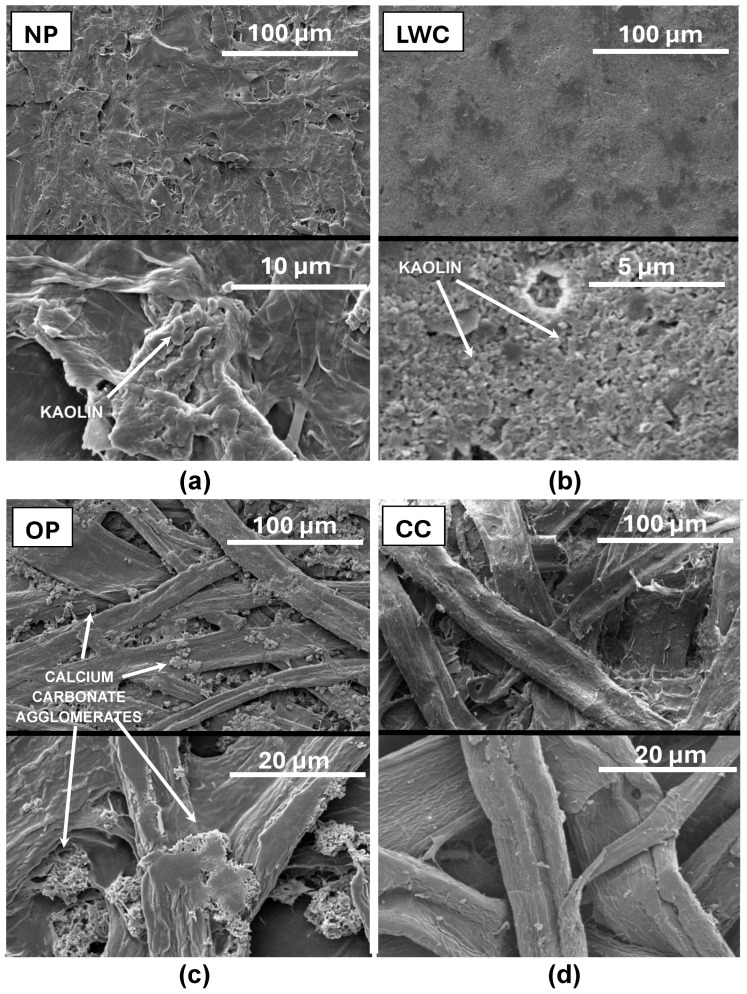
SEM images of (**a**) NP, (**b**) LWC, (**c**) OP, and (**d**) CC.

The SEM image of the CC shows clear intertwining cellulose fibers with diameters of 10–50 μm (Figure 2d).

Some of the fibers were damaged, which may indicate that a high content of recycled paper was subjected to multiple mechanical processing steps. The compartments between the fibers were poorly filled, which may point to a low content of mineral fillers. The surface of the CC was well developed.

The SEM image of the OP largely resembles that of the CC (Figure 2c,d). The material mainly consisted of densely intertwined cellulose fibers, with 10–50 μm in width. However, the OP structure seems to be more compact than that of the CC. In addition, the compartments between the fibers contain numerous agglomerates of calcium carbonate (calcite), which is typical of this material and serves as a filler [58].

The SEM image of the NP shows the outlines of cellulose fibers; however, they are not as numerous as in the OP or CC (Figure 2a). Most of the NP fibers are short and damaged, which is presumably an outcome of mechanical defibrination (during wood pulp production) [59]. Structures characteristic of kaolin can be noticed on the NP surface [60]. Fragments of cellulose fibers and lignin mass seem to be strongly compressed, which makes the material’s structure seem smoother and less porous compared to the OP and CC.

In turn, it is difficult to notice any cellulose fibers on the LWC surface (Figure 2b). This is due to the mineral coating of the paper. The structure of the coat visible on the SEM image is characteristic of the kaolin pigment coat (typical of the “gloss paper”) [61]. The LWC featured the most homogenous and smooth surface among all the tested sorbents.

#### 2.1.3. Analysis of Specific Surface Area and Porosity

Surface and porosity parameters of the waste paper sorbents are presented in Table 1. The specific surface area and pore size of the sorbents increased in the following order: NP < CC < OP < LCW. The relatively small surface area of the NP may be due to the specific production method, entailing strong pressing of the material. The LCW possessing the largest specific surface area may be attributed to its kaolin coating (Figure 2b) because, presumably, this mineral has a substantially larger surface area and porosity compared to cellulose fibers. The mean pore sizes of the analyzed sorbents were quite similar and ranged from 15.3 nm for the OP to 25.0 nm for the NP (Table 1).

### 2.2. Effectiveness of Aqueous Solution Coloration by NP, LWC, OP, and CC

**Figure 3:**
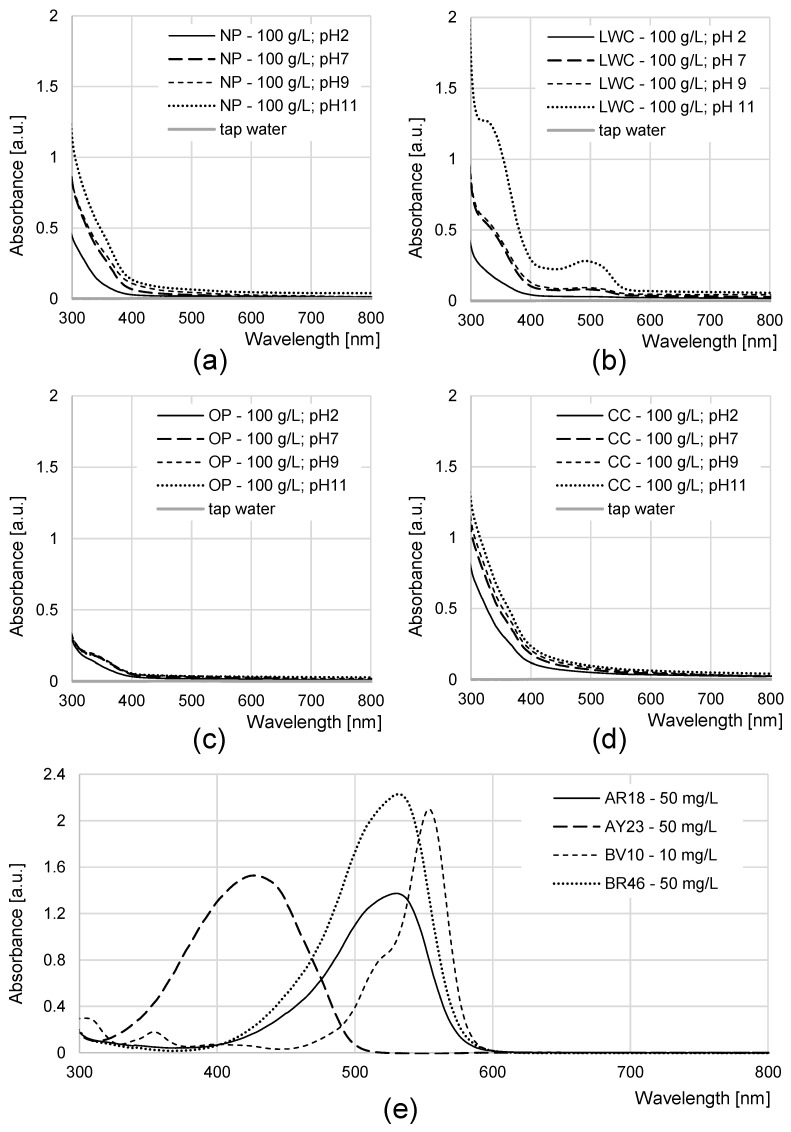
UV-VIS spectra of the aqueous solutions after 72 h contact with (**a**) NP, (**b**) LWC, (**c**) OP, and (**d**) CC, used at a dose of 100 g/L. For comparison, (**e**) spectra of the aqueous solutions of the analyzed dyes (pH 7).

The water coloration effectiveness of the analyzed sorbents was determined in order to identify the likelihood of colored contamination of the treated solutions by the waste paper materials. Figure 3a–d presents UV-VIS spectra of the aqueous solutions with various initial pH values (pH 2–11) after 72 h of contact with the NP, LWC, OP, and CC. A very high dose (100 g/L) of the waste paper materials was deliberately used in this experiment in order to increase the likelihood of detecting colored substances that underwent desorption and migrated to the water.

The UV-VIS spectra of the aqueous solutions exposed to contact with the LWC (pH 7–11) were the only ones characterized by a very broad peak present in the visible light range (Figure 3b). This peak appears at 490 nm and indicates the presence of a colored substance in the solution. It is the highest in the case of the most alkaline solution (initial pH 11), while it is absent in the spectrum of the solution with the lowest initial pH (pH 2). The high effectiveness of dye release at a high pH may point to the base nature of the colored substance (dye or pigment). The absorbance values obtained for the peak at 490 nm (≤0.25 a.u.) are low compared to the absorbance values of the peaks appearing at the λ_max_ of dyes with concentrations of 10–50 mg/L (Figure 3e), which suggests that the amounts of the colored substance potentially released by the LWC are rather negligible.

The spectra of the solutions exposed to contact with the other types of waste paper (NP, OP, and CC) showed no peaks that would indicate the presence of colored substances (dyes/pigments). It may, therefore, be hypothesized that the dyes and pigments contained in these materials, e.g., in ink or paints, were permanently bound to the paper and did not desorb over the course of this study.

The spectra of the solutions exposed to contact with the NP, LWC, and CC reveal wide absorption bands, beginning at <600 nm and increasing in intensity as wavelength decreases (Figure 3a,b,d). They are ascribed to lignin present in the solutions [62]. The absorption values of these bands depend on the solution pH, and they increase in the following order of pH values: pH 2 < pH 7 < pH 9 < pH 11. This stems from the fact that at pH > 7, lignin contained in the paper dissolves and pervades to the solution. The effectiveness of this process increases as pH increases in the system [63,64], which results in a high lignin concentration and, therefore, higher absorbance values.

The absorption bands of lignin visible in the spectra of the solutions in contact with the NP and LWC (Figure 3a,b) may overlap with the absorption bands of kaolin and also of calcite present in the solution. The bands of these mineral fillers begin at <400 nm and, as in the case of lignin, their intensity increases as wavenumber decreases [65,66]. They are, however, clearly visible in the experimental series at pH 2 (Figure 3a,b). The spectra of these solutions show no lignin absorption band because lignin does not dissolve in an acidic environment and, resultantly, does not migrate from paper to the solution.

The absorption bands of calcite are very clearly visible on the spectra of all the solutions exposed to contact with the OP (Figure 3c). OP is a woodless material which does not contaminate solutions with dissolved lignin. The intensities of calcite absorption bands in the spectra of solutions of pH values 2–11 are very similar (Figure 3c), suggesting a relatively weak effect of pH on the effectiveness of calcite release from waste paper to the solution.

In the spectra of the solutions exposed to contact with the CC (Figure 3d), the absorption bands of lignin and calcite may also overlap with the absorption band of starch because CC is produced using starch glue. The absorption band of this polysaccharide begins at the wavelength of <700 nm and its intensity increases as wavenumber decreases [67]. This observation explains the relatively high absorbance value of the band at <700 nm in the spectrum of the solution with pH 2 (Figure 3d).

The analyzed sorbents were observed to affect the color of the water that they had contact with (Table 2). The “color of water” is related to its transparency and depends on the contents of various substances capable of absorbing light radiation (both selective and non-selective absorption). Hence, water color may be developed upon the influence of not only dyes but also other organic and inorganic compounds that appear in dissolved form or as suspensions. Color is usually expressed in Hazen units (mg Pt-Co/L). It may, therefore, be concluded that the color of the analyzed aqueous solutions was affected by the total amount of substances released from the waste materials, like lignin, mineral fillers (calcite, kaolin), glue components (e.g., starch), and various dissolved or suspended contaminants.

Color intensity of the solutions exposed to contact with NP, LWC, and CC depended on pH and increased in the following order of pH values: pH 2 < pH 7 < pH 9 < pH 11 (Table 2). This increase was due to lignin concentration, and these values followed the same order as already mentioned in this section. Dissolved lignin imparted a characteristic brown color on the solution [64]. Along with lignin concentration increase, the solution became less transparent, which resulted in its successively higher color values.

At the initial pH values of 2–9, the CC was found to cause the most intense water coloration (Table 2). Presumably, the color value of water in contact with the CC was affected by starch and calcite released from this material, and also by the released lignin at pH > 9.

At pH 11, the highest effectiveness of water coloration was found for the LWC, presumably due to the fact that it possesses a colored substance capable of desorption. The effectiveness of LWC release was the highest in the strongly alkaline environment (pH 11), as is evident in Figure 3b (peak at 490 nm).

The OP material was revealed to have the lowest effectiveness of solution coloration in a broad range of pH values (pH 2–11) (Table 2). As already mentioned, it is woodless paper; hence, the lack of lignin (or its negligible amounts) in this material was reflected in its weak water coloration in the alkaline environment. Dyes or pigments in the form of ink bound to the material did not undergo any noticeable desorption, as shown in Figure 3c. In addition, the analyzed OP had no glues in its composition. Therefore, the major factor affecting the color of the solution in contact with this material was, most likely, calcite (filler of OP) released to the solution.

Generally, the effectiveness of water dyeing by the analyzed sorbents was not high. After 72 h of sorbent contact with the solutions (dose of 10 g/L), the maximal color value did not exceed 100 mg Pt-Co/L in any of the experimental series. In the case of the OP, it did not even exceed 15 mg Pt-Co/L, regardless of the initial pH of the water. For comparison, the color value determined for tap water available at the laboratory reached 3.13 mg Pt-Co/L over the course of this study, while the value recommended for tap water is <15 mg Pt-Co/L.

To recapitulate, this preliminary study demonstrates that the effectiveness of colored contamination of the aqueous solutions by the tested materials was very low. It may, therefore, be assumed that waste papers used as sorbent will not pose any severe threat to the aquatic environment.

Despite the very weak effect of the NP, LWC, OP, and CC on the color of the analyzed aqueous solutions, it will be still considered in determinations of their dye sorption capability (as mentioned in comments in Section 4.3, Section 4.4 and Section 4.5).

### 2.3. Effect of pH on the Effectiveness of Dye Sorption on NP, LWC, OP, and CC

**Figure 4:**
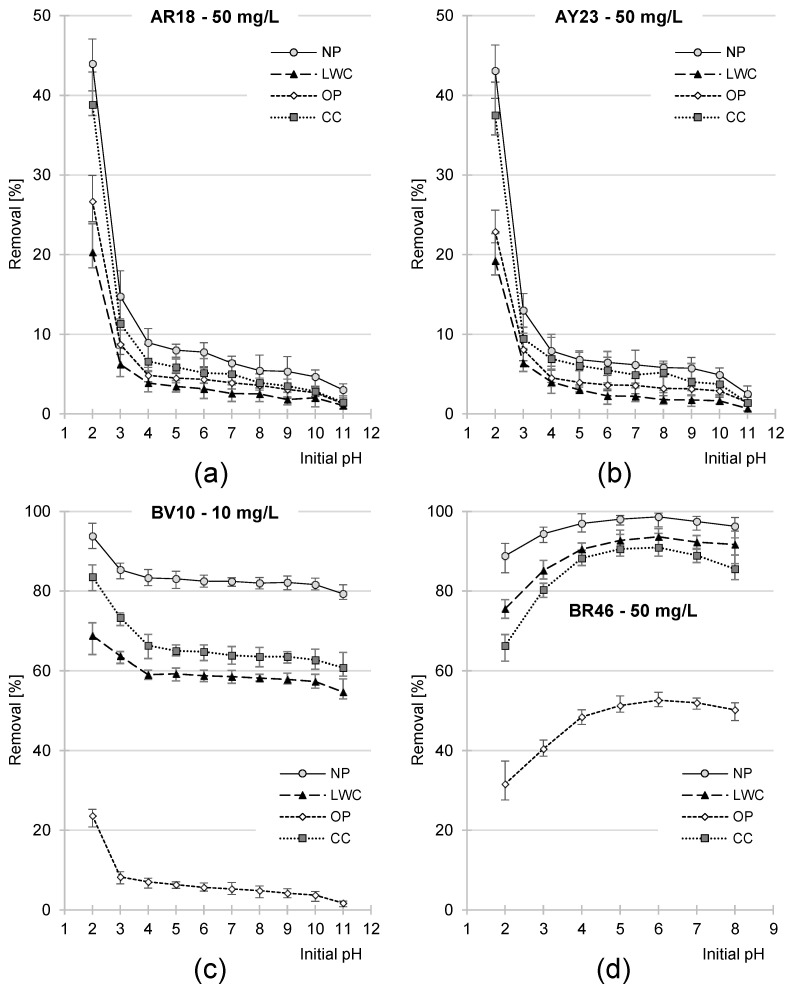
Effect of pH on the sorption effectiveness of (**a**) AR18, (**b**) AY23, (**c**) BV10, and (**d**) BR46 onto NP, LCW, OP, and CC (10 g/L dose).

The sorption effectiveness of the anionic dyes (AR18, AY23) on all the tested waste paper sorbents (NP, LWC, OP, and CC) was the highest at pH 2 and decreased as pH increased, reaching the lowest value at pH 11 (Figure 4a,b). The greatest decrease in the sorption effectiveness of these dyes was noted at a pH range of pH 2–3, whereas the effectiveness remained relatively low at pH 4–10.

The high sorption effectiveness of the analyzed anionic dyes on the tested sorbents at a low pH was due to protonation of the functional groups of polysaccharides (cellulose and hemicellulose) and lignin in the acidic environment. In the case of both holocelluloses and lignin, these are usually the hydroxyl groups that undergo protonation.

-OH + H_3_O^+^ → -OH_2_^+^ + H_2_O

Positively charged hydroxyl groups electrostatically attracted the anions of dyes present in the solution, thereby increasing the effectiveness of their sorption.

The protonation effectiveness of functional groups largely depends on the concentration of hydronium ions in the solution, i.e., on the pH of the system. As the pH increased, the number of positively charged functional groups decreased on the sorbents’ surface, which was reflected in the diminishing sorption effectiveness of the anionic dyes.

A characteristic feature of hydroxyl functional groups is their effective protonation only in a strongly acidic environment (<pH 3). This explains the sharp decrease noted in the sorption effectiveness of AR18 and AY23 at pH 2–3 (Figure 4a,b).

In a pH range of pH 4–10, sorption most likely followed the mechanism of hydrogen bond formation, e.g., bonds between atoms of nitrogen or oxygen present in the dye’s structure and hydrogen atoms of the hydroxyl groups of the sorbents. Due to the very low dye sorption effectiveness in this pH range, this mechanism was, presumably, of marginal importance. In alkaline conditions, the sorbents’ surface could attain a negative charge, e.g., as a result of deprotonation of its hydroxyl groups.

-OH + OH^−^ → -O^−^ + H_2_O

At high pH, the negatively charged functional groups of the sorbents could electrostatically repel the anions of dyes, which inhibited their sorption. The number of deprotonated functional groups reflected in the total negative charge on the sorbents’ surface increased as pH increased, which explains why the highest sorption effectiveness of anionic dyes occurred at the highest pH tested (Figure 4a,b).

A very similar pH effect on dye binding effectiveness was also noted in studies addressing AR18 sorption on rapeseed hulls [68], sunflower seed hulls [69], and carbonized biomass of common reed. In the case of AY23, the positive impact of low pH on its sorption effectiveness was observed in experiments with sawdust [70], cotton fibers [71], chitin [72], and activated carbons [73,74,75].

As in the case of the anionic dyes, the sorption effectiveness of the cationic dye BV10 on the NP, LWC, OP, and CC was the highest at pH 2 and decreased as pH increased (Figure 4c). The greatest decrease in BV10 sorption effectiveness was noted in the initial pH range of pH 2–4, whereas at pH 4–10 its sorption effectiveness remained similar.

As observed for the anionic dyes, the impact of pH on BV10 sorption may be due to its carboxyl functional group, which easily undergoes deprotonation, generating a local negative charge. Despite its generally cationic nature, BV10 may act as an anionic dye at low pH values due to this group. This phenomenon has been confirmed in other studies examining its sorption by unconventional sorbents, like champignon biomass [76] or powdered coffee [77]. At higher pH values (pH 4–10), BV10 sorption on the tested sorbents may have been largely affected by hydrogen bonds.

The sorption effectiveness of the cationic dye BR46 on the analyzed sorbents was the lowest at pH 2 and increased as pH increased, peaking at pH 6 (Figure 4d). Successive pH increase in the system caused a negligible decrease in its sorption effectiveness.

BR46 is a typical representative of cationic dyes. Although the analyzed waste papers did not possess typical functional alkaline groups, they proved to bind quite well. The sorption of BR46 on the NP, LWC, OP, and CC could have proceeded mainly via hydrogen bonds generated between hydrogen atoms of the hydroxyl groups of the sorbents and the nitrogen atoms of the dye. The formation of hydrogen bonds between the oxygen atoms the sorbents’ hydroxyl and carbonyl groups and the hydrogen atoms of the BR46 functional groups is also likely.

The lowest BR46 sorption effectiveness on the tested sorbents was recorded at pH (pH 2) due to strong electrostatic repulsion between the positively charged surfaces of the sorbents and the dye cations. The total positive charge on each sorbent’s surface decreased along with pH increase, resulting in the boosted effectiveness of BR46 sorption. At pH > 7, its sorption on waste paper sorbents may have been impaired by competition with sodium cations for active centers available on the sorbent’s surface.

A characteristic feature of BR46 aqueous solutions is their discoloration under alkaline conditions (pH > 8). This observation was also confirmed in our previous study analyzing its sorption on bird feathers [78] and mealworm exoskeletons [79]. The results of the spectrophotometric analyses of BR46 solutions from the experimental series performed at pH 9–11 could, therefore, be wrongly interpreted. For this reason, Figure 4d does not contain any of the experimental data achieved in this pH range.

A very similar pH effect on BR46 sorption effectiveness was also noted in studies examining its removal on citrus fruit peels [80], coconut shells [81], and spent coffee leaves and grains [35].

The impact of pH on sorption effectiveness was determined by dye type, whereas for each tested dye, the effect of pH on the sorption of the NP, LWC, OP, and CC was similar (Figure 4). This observation suggests that the chemical nature of all waste paper-based materials is very similar, although they differed in their dye sorption effectiveness. In the case of the anionic dyes AR18 and AY23, effectiveness increased in the following order: LWC < OP < CC < NP (Figure 4a,b). As in the case of the anionic dyes, the sorption of cationic dyes was the most effective with the NP. In turn, the OP proved to be the least effective sorbent of BV10 and BR46 (Figure 4c,d). The differences observed in the sorption effectiveness of the analyzed sorbents are, presumably, due to their chemical compositions.

**Figure 5:**
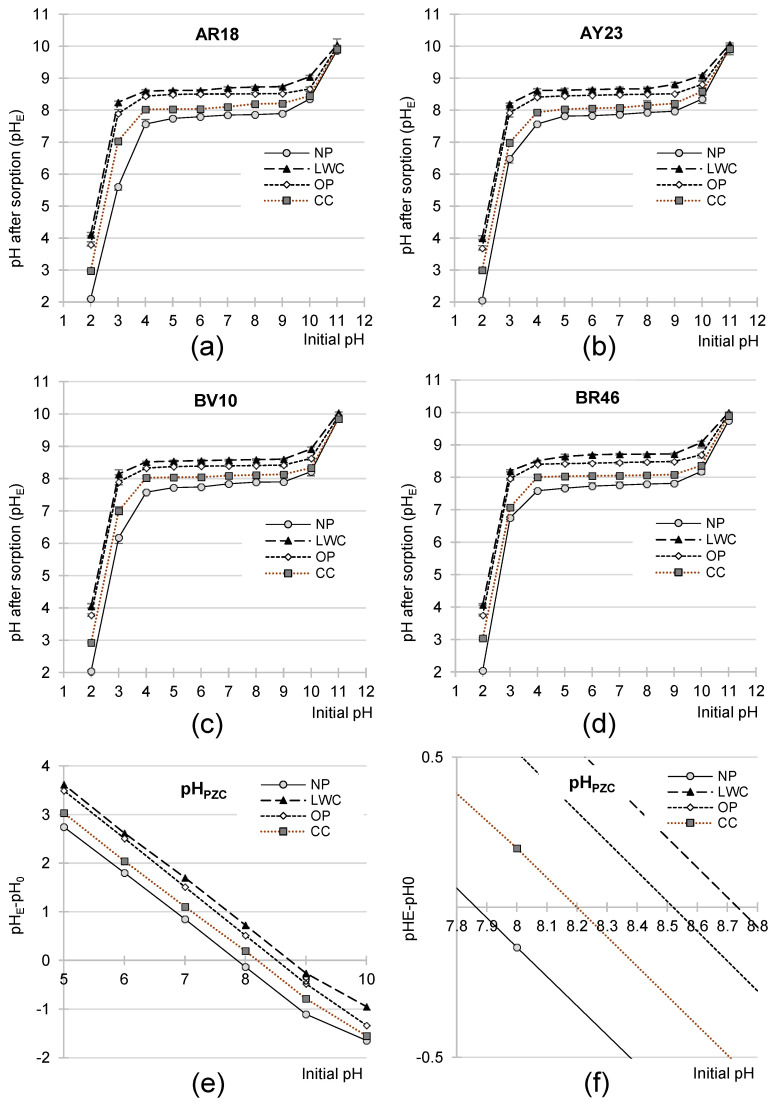
Changes in pH values of the solutions during sorption of (**a**) AR18, (**b**) AY23), (**c**) BV10, and (**d**) BR46 dyes onto NP, LCW, OP, and CC (10 g/L dose). (**e**,**f**) pH_PZC_ of the tested sorbents determined with the “drift” method.

The analyzed waste paper-based sorbents were found to modify the pH values of dye solutions during sorption (Figure 5a–d). At the initial solution pH range of pH 4–9, the pH values measured after 120 min of sorption fell within the following ranges: pH 7.57–7.95 for NP, pH 8.62–8.81 for LWC, pH 8.41–8.54 for OP, and 7.94–8.22 for CC. The changes in solution pH triggered by contact with the waste materials are a typical phenomenon for sorbents possessing functional groups capable of ionization. The mechanism of this process is known and stems from the possibility of the sorbents’ surface accepting protons under conditions of low pH, or of their donation at high pH. Solutions in systems always tend to reach a pH value approximating sorbent pH_PZC_. The pH_PZC_ values determined with the “drift” method reached pH 7.85 for NP, pH 8.74 for LWC, pH 8.52 for OP, and 8.20 for CC (Figure 5e,f).

Usually, higher pH_PZC_ values of sorbents (pH_PZC_ > 7) indicate the presence of typically cationic functional groups (e.g., amine groups) in a material’s structure and, thus, their good sorption capabilities towards anionic dyes. In turn, a pH_PZC_ < 7 points to the presence anionic groups (e.g., carboxyl groups) and generally to an alkaline nature of the sorbents, both of which facilitate cationic dye sorption. In the present study, however, there was no clear correlation between the pH_PZC_ values of the waste paper sorbents and their capabilities of dye sorption. This lack of correlation may be explained by some effect of the fillers contained in the waste papers (like calcite, CaCO_3_) on the changes in the solutions’ pH and, thus, pH_PZC_ values. Calcium carbonate released to the solutions could have increased the pH of the system, which could have elicited significant effects, especially in the experimental series with the calcite-rich OP. The relatively highly pH_PZC_ of the LWC (pH_PZC_ = 8.74) was due to its kaolin pigment coating. During its production, an alkaline pH (minimum pH 8.5) [82,83] is deliberately maintained to preclude calcium carbonate degradation.

The successive study stages were conducted at sorption pH values optimal for the individual dyes, i.e., pH 2 for AR18, AY23, and BV10, and pH 6 for BR46; the results are described in Section 2.4 and Section 2.5.

### 2.4. Kinetics of Dye Sorption on NP, LWC, OP, and CC

**Figure 6:**
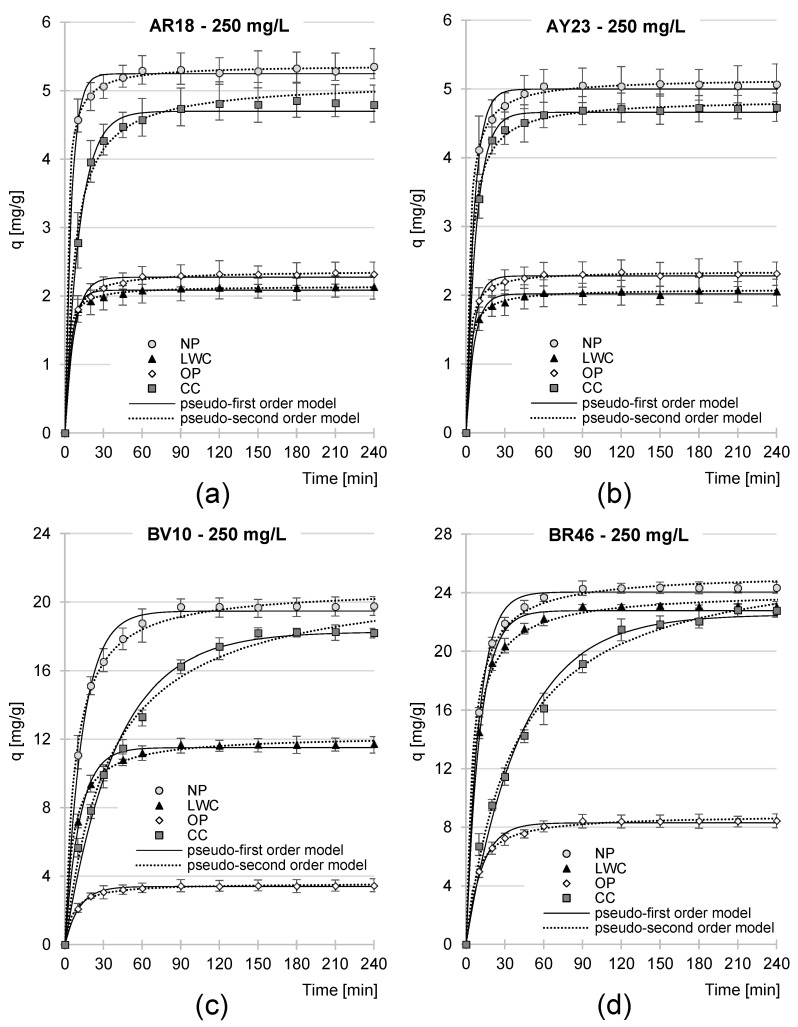
Sorption kinetics of (**a**) AR18, (**b**) AY23, (**c**) BV10, and (**d**) BR46 onto NP, LCW, OP, CC (10 g/L dose) (average + range). Pseudo-first-order model and pseudo-second-order model.

The sorption equilibrium time of the anionic dyes, AR18 and AY23, on the analyzed sorbents depended on their initial concentration, ranging from 60 to 90 min for the NP, LWC, and OP, as well as from 120 to 150 min for the CC (Table 3, Figure 6 and Figure S1). The sorption intensity of these dyes was the highest at the beginning of the process; the amounts of anionic dyes bound with the sorbents ranged from 75.8 to 91.9% of the q_e_ value (q_e_—the amount of dye sorbed at the equilibrium state) as early as after first 20 min of sorption (Figure 6 and Figure S1).

The dye sorption equilibrium times of 60–90 min (i.e., the same as those achieved for NP, LWC, and OP) were also reported in studies investigating AR18 sorption on seaweed biomass (60 min) [84], activated carbon from carrot biomass (80 min) [85], and sunflower seed hulls (90 min) [69], and in the case of AY23 on activated carbon from coconut shells (60 min) [81], sawdust (70 min) [70], and activated carbon from cassava (90 min) [75]. In turn, equilibrium times reaching 120–150 min (like for the CC in the present study) were also noted during AR18 removal on carboxymethylcellulose (120 min) [86] and activated carbon from poplar wood [68,87], as well as during AY23 sorption onto commercial activated carbon (120 min) [88].

The sorption times of the cationic dyes on the NP, LWC, and OP also depended on their concentration and ranged from 60 to 90 min for BR46 and from 90 to 120 min for BV10. Substantially longer sorption times were determined in the experimental series with the CC, where the equilibrium sorption time of the cationic dyes ranged from 150 to 210 min. As in the case of the anionic dyes, the effectiveness of BV10 and BR46 binding on the tested sorbents was the most intensive at the beginning of the sorption process. After 20 min, the amounts of cationic dyes bound on the NP, LWC, and OP reached 70.0–91.3% of q_e_, and 41.7–45.7% q_e_ for the CC.

The 60–120 min equilibrium sorption time of the cationic dyes (typical of NP, LWC, and OP) was also obtained in studies addressing BV10 sorption on *Calotropis procera* leaves (60 min) [89], coconut fibers (90 min) [90], and chitin from mealworm exoskeletons (120 min) [91], as well as in studies examining BR46 binding on sugar cane biomass (60 min) [92], bone meal (90 min) [93], and beech sawdust (120 min) [94]. In turn, longer sorption times, within the range of 150–210 min (like in the case of CC), were noted during BV10 binding on carbonized coconut fibers (150 min), coffee grains (180 min) [77], and champignon biomass (210 min) [76]; during BR45 sorption on, these times were noted with fir sawdust (150 min) [95], mealworm exoskeletons (180 min) [79], and hen feathers (210 min) [78].

Generally, the shorter dye sorption times that are achieved at their higher initial concentrations may be due to the greater likelihood of collisions of dye molecules with the sorption centers of waste paper materials. Faster saturation of the active sites of the sorbents resulted in a faster achievement of equilibrium in the system.

The noticeably longer dye sorption times recorded in the experimental series with the CC may stem from the significantly greater thickness of the sheets of this material and its grammage compared to the NP, LWC or OP (Table 8, Section 3.1). The greater thickness of this sorbent may have strongly impaired the dyes’ access to the active sites in its deeper layers, which substantially elongated the sorption process.

Generally, the longer sorption equilibrium times of BV10 compared to BR46 may be due to the higher molecular weight of Basic Violet 10, as penetration of its large molecules to the active sites located in deeper layers of sorbents was more difficult, which extended sorption time.

The kinetics of dye sorption on the analyzed sorbents was described with the pseudo-first- and pseudo-second-order models. In each experimental series, regardless of dye type and concentration and regardless of sorbent type, the experimental data are best described with the pseudo-second-order model (Table 3, Figure 6, Figures S1 and S2), which is typical of the sorption of organic dyes onto biosorbents.

**Figure 7:**
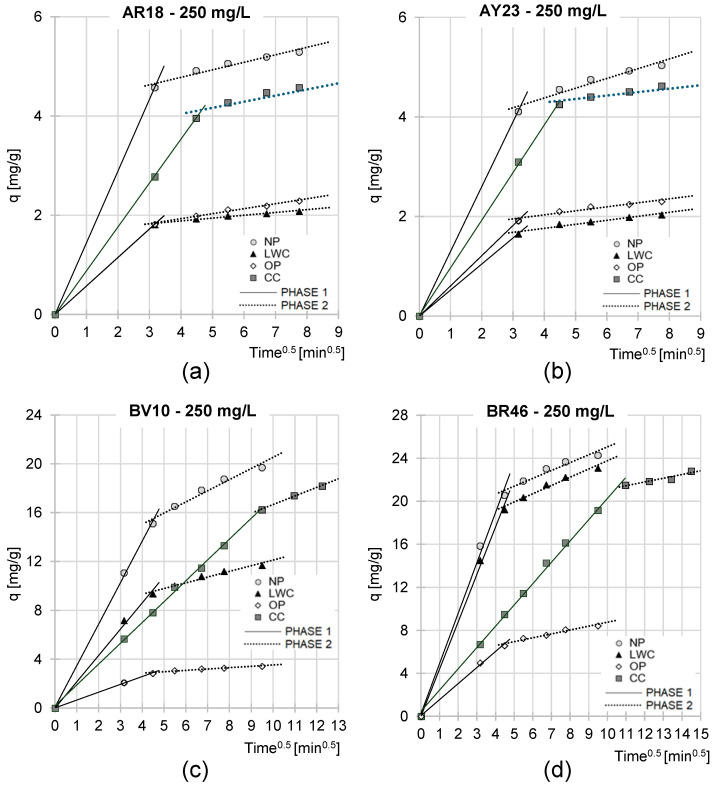
Intraparticular diffusion model of the sorption of (**a**) AR18, (**b**) AY23, (**c**) BV10, and (**d**) BR46 onto NP, LCW, OP, and CC (10 g/L dose).

Experimental data from analyses of dye sorption kinetics were also described using the intraparticular diffusion model (Table 4, Figure 7, Figures S3 and S4). Analysis of the data presented in Figure 7 indicates that over all the experimental series, dye sorption on the examined sorbents proceeded in two main phases.

The first phase of sorption was highly intense and short. Presumably, during this phase, dye molecules diffused from the solution onto the sorbent’s surface and occupied its most available sorption centers. Once the most active sites had been occupied on the material’s surface, the second phase began. During this phase, dye molecules most likely occupied active sites located in deeper layers of the sorbent. Due to the increasingly impaired access to the sorption centers and high competitiveness between the dye molecules for the depleting free centers, the course of sorption in this phase was significantly less intensive and generally lasted much longer compared to the first phase. Once the last free active sites available in the sorbent’s structure had been occupied, the system entered into the state of equilibrium.

Somehow, different observations were made during the sorption of the cationic dyes BV10 and BR46 on the CC, where phase I was exceptionally long and even longer than phase II (Figure 7 and Figure S4, Table 4). This finding may be due to the high grammage of this material, facilitating access to active sites located in its deeper layers. The specific combination of these properties was reflected in the high number of sorption centers easily available to BV10 and BR46. The higher number of active sites could, theoretically, have resulted in a longer duration of phase I. Such an outcome was not observed in the cases of the anionic dyes, probably due to their sorption different mechanisms and also to a significantly lower number of active centers (groups -OH_2_^+^) available to AR18 and AY23 on CC’s surface.

The values of k_2_ and q_e_ as well as the k_d1_ and k_d2_ constants determined from the pseudo-second-order and intraparticular diffusion models suggest relatively strong correlations between dye concentration and sorption intensity for all the dyes and sorbents tested (Table 3 and Table 4).

The sorption kinetics of the anionic dyes on the tested sorbents was similar (Figure 6a,b and Figure 7a,b; Table 3 and Table 4), most likely due to the similar molecular weights of AR18 and AY23 and their identical number of acidic functional groups. In the case of the cationic dyes, the higher rate of BR46 sorption, compared to BV10 (Figure 6c,d and Figure 7c,d; Table 3 and Table 4), might have been due to its lower molecular weight. Smaller BR46 molecules could more easily diffuse to the sorption centers located deeper in the sorbent’s layers, which—as already mentioned—could result in shorter but more effective sorption.

Analysis of the data presented in Figure 6 and Figure 7 shows that the sorption rate of the anionic dyes AR18 and AY23 on the tested sorbents increased in the order LWC < OP < CC < NP, whereas that of the cationic dyes increased in the following order: OP < LWC < CC < NP. These observations are generally consistent with the orders of dye sorption rate increases determined in Section 2.3. (Figure 5). As already mentioned, these findings are related to the chemical composition of the sorbents, which are more extensively described in Section 2.5.

There were no significant correlations between the measured specific surface area and porosity of the paper waste materials (Table 1) and their dye sorption kinetics, which may be ascribed to the sorbents’ surface morphology modification upon contact with water.

### 2.5. Maximum Sorption Capacity of NP, LWC, OP, and CC

**Figure 8:**
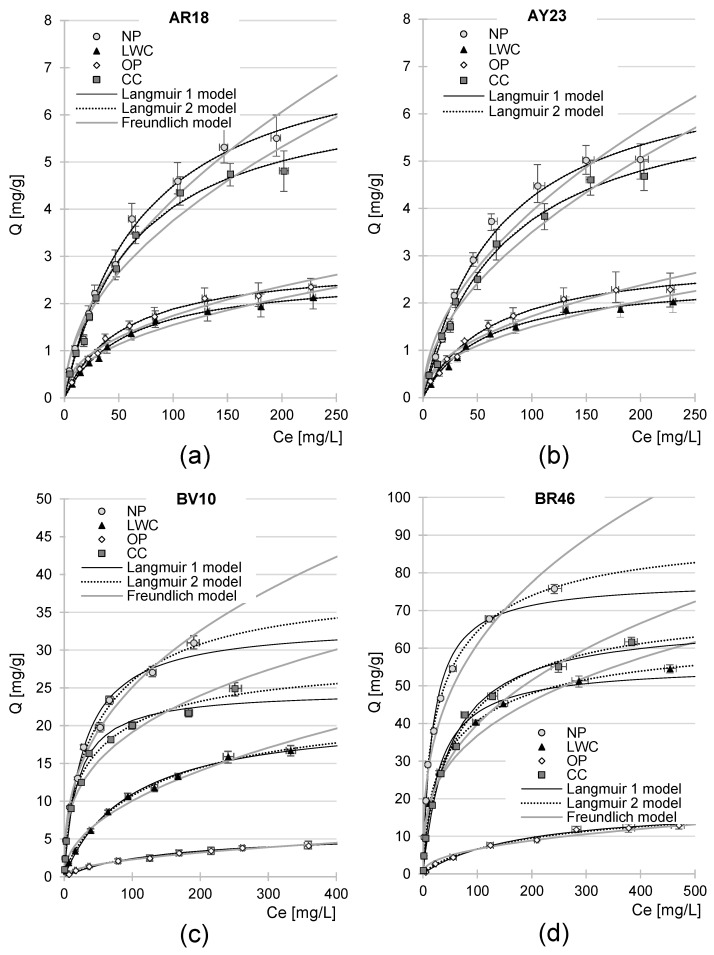
Sorption isotherms of (**a**) AR18, (**b**) AY23, (**c**) BV10, and (**d**) BR46 onto NP, LCW, OP, and CC (10 g/L dose); (average + range). Langmuir 1, Langmuir 2, and Freundlich models.

The experimental data from determinations of the maximum dye sorption capacities of the waste paper sorbents were described using popular sorption models: Langmuir 1 isotherm, Langmuir 2 isotherm, and Freundlich isotherm (Figure 8, Table 5). Analysis of the determination coefficients (R^2^) shows that the Langmuir models fitted to the experimental data better than the Freundlich model did, in all experimental series. This points to quite a specific mechanism of adsorption, where only one dye molecule can attach to one active center on a sorbent’s surface. In consequence, dye molecules form a “monolayer” on the sorbent’s surface. In addition, the bond formed between the sorbate and the active site is not permanent, and dye molecules can exchange their bound sorption centers between one another and also change their position within the monolayer.

In all the experimental series with the anionic dyes, the constants determined from the Langmuir 1 model (K_C_, Q_max_) had the same numeric values as the constants determined from the Langmuir 2 model (K_1_/K_2_, Q_max_) (Table 5). This finding suggests that only one type of sorption center played a key role in AR18 and AY23 sorption on the tested sorbents. Most likely, these were the hydroxyl groups protonated on the sorbent’s surface, which electrostatically interacted with the acidic groups of the anionic dyes.

The experimental data achieved in this series with the cationic dyes were better described by the Langmuir 2 model than the Langmuir 1 model (Table 5), which indicates that at least two types of active sites played a major role in their sorption on the analyzed waste paper materials. In the case of Basic Red 46, it was probably hydrogen atoms of the sorbent’s hydroxyl groups that formed strong hydrogen bonds with nitrogen atoms of the dye, in addition to oxygen atoms of the hydroxyl and carbonyl groups of the sorbents that formed weak hydrogen bonds with nitrogen atoms present in the functional groups of BR46. π–π (pi–pi) interactions between the aromatic rings of lignin and dye also likely occurred for the sorbents containing lignin (NP, LWC, and CC) [96]. In the case of BV10, apart from the active sites capable of forming hydrogen bridges and π–π interactions, hydroxyl functional groups also played an important role in its sorption, as they electrostatically interacted with its ionized carboxyl group.

The maximum sorption capacity of the analyzed waste paper sorbents on the anionic dyes was relatively low. The NP proved to be the most effective sorbent of AR18 and AY23, with maximum sorption capacities of 7.77 mg/g and 7.20 mg/g, respectively, whereas the LWC turned out to be the least effective in this respect, with maximum sorption capacities of barely 2.64 mg/g and 2.54 mg/g, respectively.

The sorption effectiveness of the anionic dyes on the analyzed waste paper sorbents decreased in the order NP > CC > OP > LWC, which may have been due to the coeffect of several important factors.

An important parameter of the waste paper sorbents is their lignin–to–holocellulose (cellulose, hemicellulose) ratio. Their chemical structure includes a few types of hydroxyl functional groups, which differ in their reactivity. Lignin possesses primary and secondary aliphatic hydroxyl groups, which are constituents of phenyl groups. In turn, cellulose and hemicellulose contain primary aliphatic hydroxyl groups (C6-OH) as well as two types of secondary hydroxyl groups, linked directly with the saccharide ring (C2-OH and C3-OH) [97]. The aliphatic hydroxyl groups, both the primary and secondary ones, are generally more reactive and more susceptible to ionization than the hydroxyl groups bound directly to the saccharide or aromatic rings [97,98]. The ratio of aliphatic -OH groups to the -OH groups bound to the chemical ring is higher in lignin than in cellulose, which suggests that lignin may potentially have a higher number of protonated hydroxyl groups than cellulose and, thus, that it may exhibit a higher capability for AR18 and AY23 sorption. Therefore, the lignin content of the sorbent may significantly boost the sorption effectiveness of anionic dyes. Among the analyzed sorbents, the highest lignin–to–holocelluloses ratio was found in the NP (Table 8, Section 3.1), which may explain its higher dye sorption effectiveness. Second after the NP in this respect was the CC, which is consistent with the determined order of sorbent effectiveness in AR18 and AY23 binding (NP > CC > OP > LWC) and also confirms the theory of the positive impact of lignin on the sorption effectiveness of these dyes. In turn, the OP represents a material free of lignin (or possessing its negligible amounts, Table 8), which—according to the presented theory—may explain its remarkably poorer sorption properties towards AR18 and AY23 compared to the NP and CC. Despite possessing lignin in a quantity similar to that found in the CC, LCW turned out to be the worst sorbent of anionic dyes. Its typical feature of a kaolin pigment coating and, presumably, kaolin could significantly impair the access to sorption centers present in lignin and cellulose, thereby severely diminishing the sorption capability of this material. In addition, kaolin is a mineral material with a provenly very low capability for binding anionic dyes [99]. This explains the low LWC usability for removing AR18 and AY23 from aqueous solutions. The better sorption capability of the OP towards anionic dyes compared to the LWC could also be due to its high calcite content, which exhibits far better potential for anionic dye sorption than kaolin [100].

The analyzed sorbents showed significantly higher sorption capacities towards cationic dyes than anionic dyes. The K_1_ values determined from the Langmuir 2 model also prove the higher affinity of BV10 and BR46 to the active sorption centers, compared to AR18 and AY23. The NP turned out to be the best sorbent of BV10 and BR46, yielding sorption capacities of 33.53 mg/g and 78.01 mg/g, respectively. In turn, dye sorption was the least effective with the OP, which had Q_max_ values of 5.89 mg BV10/g and 18.41 mg BR46/g (Table 5).

The effectiveness the waste paper sorbents’ cationic dye sorption decreased in the order NP > CC > LWC > OP, which perfectly matches the lignin contents of these materials, suggesting that—like in the case of anionic dyes—lignin strongly affects their sorption process. As mentioned earlier, the mechanism of cationic dye sorption obviously differs from that of anionic dyes. Lignin has a more complex chemical structure compared to cellulose or hemicellulose. It is composed of heterogeneously cross-linked lignols, which are derivatives of phenylpropane. It contains both aliphatic and aromatic units in its structure and, besides hydroxyl functional groups, it possesses carbonyl, methoxyl, and phenolic groups. The number of lignin active sites able to form hydrogen bonds with atoms of nitrogen and hydrogen in cationic dyes is generally higher than in cellulose or hemicellulose. In addition, its aromatic structures may enter into π–π (pi–pi) reactions with BV10 and BR46 rings, which may explain the relatively strong correlation between the lignin content of the sorbents and their cationic dye sorption effectiveness. The substantially poorer sorption capability of the OP towards BV10 and BR46 compared to the other analyzed waste paper sorbents may stem not only from the lack of lignin (or its minute content) but also from its high content of calcite, which is known for its poor capability in the sorption of cationic dyes.

The remarkably poorer potential of the waste paper materials for the sorption of AR18 and AY23 compared to BV10 and BR46 may result from the fact that they have no typically alkaline functional groups (e.g., amine or acetamide groups), which represent the best type of sorption centers in anionic dyes.

The lower sorption effectiveness of BV10 compared to BR46 may, in turn, be due to the higher molecular weight of the first, hampering the attachment of its large molecules to the sorption centers located in deeper layers of the sorbent. The other reason could be its acidic functional group (-COOH), which is not typical for a cationic dye. Owing to this group, BV10 possessed two opposite electrical charges, which might have electrostatically disturbed its molecules’ interactions with the sorbent’s surface.

There was no clear correlation between each sorbent’s surface morphology (specific surface area, porosity, pore diameter—Table 1) and its maximum sorption capacity. It is likely that the structure of waste paper sorbents undergoes certain modifications when in contact with water. Presumably, the distance between cellulose fibers increases in soaked paper, resulting in greater surface area, porosity, and, consequently, in the better sorption capabilities of these materials. The extent of sorbent structure loosening upon contact with water may depend on material type. However, precise measurements of the specific surface area and porosity of materials in an aquatic environment are difficult to perform.

Table 6 and Table 7 compare the sorption capacities of the waste paper sorbents analyzed in this study with those of other unconventional sorbents and activated carbons (literature data).

The anionic dye sorption capabilities of waste paper materials are relatively small compared to selected unconventional solvents. The sorption capacities of NP, LWC, OP, and CC towards AR18 and AY23 are substantially lower than materials based on biocarbons and activated carbons (Table 6). Materials based on plant biomass, like seaweed, rapeseed hulls, compost, or soybean pomace, as well as materials of animal origin, like chitin or chitosan flakes, also turned out to be better sorbents of anionic dyes. The higher sorption capacities achieved by biomass-based sorbents could be attributed to their alkaline functional groups (e.g., amine groups), which—as mentioned earlier—represent the best type of active center for anionic contaminants. The sorption capabilities of the waste paper sorbents were only higher than those of coconut shells and sunflower seed hulls, which could be due to the larger specific surface area of corrugated cardboard.

The analyzed sorbents exhibited relatively good sorption capabilities towards cationic dyes. NP, LWC, and CC all achieved higher BV10 and BR46 sorption capacities than plant biomass-based materials like sawdust, cones, crop leaves, seed hulls, nut shells, and fruit peels (Table 7). This finding confirms the theory that the sorption capabilities of biosorbents towards cationic dyes are largely determined by their lignin and cellulose contents.

The tested waste paper materials, except for the OP, turned out to be more effective sorbents of BV10 and BR46 compared to the materials of animal origin, like feathers, chitin, or chitosan. Notably, NP, LWC, and CC were equal in this respect, even in activated carbons (Table 7). As already mentioned, the substantially worse sorption capability of OP, compared to the other analyzed waste paper sorbents, could be due to its high content of calcite (CaCO_3_), which exhibits a very low capability for binding cationic contaminants.

Potentially, waste paper sorbents such as NP, LWC, and CC could be applied in the treatment of post-production waters containing cationic dyes. In selected systems designed for wastewater decolorization, they could serve as cheaper substitutes of activated carbons.

A disadvantage of waste paper-based sorbents is their potential reuse. The regeneration of used waste paper sorbents, which involves the desorption of dyes and requires the use of expensive chemical reagents. In addition, this process leads to the generation of new wastewater batches that need to be treated. Considering the low price of waste paper, which is a few to several dozen times cheaper than commercially available sorbents, the treatment of used waste paper does not seem to be economically justified. However, after sorption, waste paper sorbents can be dried and then incinerated, e.g., in heating plants, to generate thermal energy. Alternatively, used waste paper sorbents can be bio-converted and then fermented to produce biogas. Used waste paper can also be subjected to carbonization and activation processes to produce activated carbon, which can be used in wastewater treatment.

## 3. Materials

### 3.1. Analyzed Types of Waste Paper

Used newsprint (NP) was sourced from outdated Polish newspapers: “Gazeta Wyborcza”, “Rzeczpospolita”, and “Dziennik Gazeta Prawna”. The newspapers were bought on working days in Polish press points from the 1st to the 30th November 2023 (total number of purchased newspapers: n = 78).

Used lightweight coated paper (LWC), also referred to as used gloss paper, derived from outdated monthly magazines: “Wiadomości Uniwersyteckie” (Olsztyn), “Glamour” (Poland), and “Twój Styl”. The magazines were purchased in Polish press points from January to December 2023 (total number of purchased magazines: n = 36).

Used office paper (OP) derived from outdated or unnecessary documents in the A4 format, made available by the secretariat of the Department of Environmental Engineering, University of Warmia and Mazury in Olsztyn, Poland. The documents were printed from January to December 2023. A total of 1000 sheets covered with at least 60% text were selected for analyses.

Used corrugated cardboard (CC) derived from packaging and shipments delivered to the Department of Environmental Engineering, University of Warmia and Mazury in Olsztyn, Poland, from January to December 2023. Three-layer, unbleached, uncolored corrugated cardboard, without a protective coating (the most popular type of corrugated cardboard), was selected for analysis.

Table 8 presents the approximated parameters of the analyzed sorbents (literature data).

### 3.2. Dyes

This study was conducted with two anionic dyes, Acid Red 18 (AR18) and Acid Yellow 23 (AY23), and two cationic dyes, Basic Violet 10 (BV10) and Basic Red 46 (BR46). All the dyes were purchased at the Dye Producing Plant “BORUTA-ZACHEM KOLOR SA” in Zgierz (Poland). Their key parameters are listed in Table 9.

### 3.3. Chemical Reagents

- Hydrochloric acid (HCl)—37%—(solution pH correction);
- Sodium hydroxide (NaOH) > 99.9%—micropellets—(solution pH correction);
- Acetone (C_3_H_6_O, >99.5%)—cleaning the diamond crystal in the ATR attachment of the spectrometer;
- Buffer solutions (pH 4 ± 0.05/pH 7 ± 0.05/pH 10 ± 0.05)—calibration of the pH meter.
- All the chemical reagents used were purchased from POCH S.A., Gliwice, Poland, and were of p.a. (analytical purity) grade or higher.

### 3.4. Laboratory Equipment

- HI 110 pH meter (HANNA Instruments, Olsztyn, Poland)—for the measurement and correction of pH solutions;
- Laboratory shaker SK-71 (JEIO TECH, Daejeon, Republic of Korea)—for the sorption process;
- Multi-Channel Stirrer MS-53M (JEIO TECH, Daejeon, Republic of Korea)—for dye sorption analyses;
- UV-3100 PC spectrophotometer (VWR spectrophotometers, VWR International LLC., Mississauga, ON, Canada)—for determination of the concentration of dye in solutions;
- FT/IR-4700LE FT-IR Spectrometer with single reflection ATR attachment (JASCO International, Tokyo, Japan)—for the preparation of sorbent FTIR spectra;
- ASAP 2020 (Micromeritics, Norcross, GA, USA)—for measurements of porosity and surface area of the sorbent;
- Quanta 200 Scanning Electron Microscope (FEI, Eindhoven, The Netherlands) —for taking images of the sorbent surface morphology.

## 4. Methods

### 4.1. Preparation of Sorbents

As mentioned in the “Introduction” section, following the idea of “maximal cost reduction”, the waste papers used in this study were not pre-treated (purified) or modified in any way. Their preparation mainly entailed their disintegration.

All the collected copies of newspapers (NP) and monthly magazines (LWC) as well as sheets of documents (OP) were disintegrated into 2 × 15 mm pieces using an office shredder. Before the LWC magazines had been shredded, their covers were removed due to the different paper characteristics. The corrugated cardboard (CC) was cut into approximately 2 × 15 mm pieces using a paper guillotine. After disintegration, each waste paper type was mixed to homogenize the material batch. The sorbents prepared in this way were stored in air-tight polypropylene containers.

### 4.2. Determination of the Coloration Effectiveness of Aqueous Solutions by the Tested Sorbents

Portions of each sorbent (20 g dry matter (d.m.) each) were weighed into 3000 mL laboratory beakers. Next, 2000 mL of deionized water of pH 2, pH 7, pH 9, and pH 11 (water pH was adjusted with HCl or NaOH solution) were added to the beakers. The contents of the beakers were mixed, and then the beakers were protected with a parafilm and left in a dark place for 72 h. Afterwards, the samples were collected from the beakers for solution color determination in Hazen’s units (mg Pt-Co/L). Color profiles were determined in the APHA color system.

To achieve clear UV-VIS spectra of the prepared solutions, the experiment was repeated using a 10-fold-higher sorbent dose (100 g/L).

### 4.3. Determination of pH Effect on Dye Sorption Effectiveness

Portions of the sorbents (2.5 g d.m. each) were weighed into a series of 1000 mL conical flasks. Next, dye solutions (250 mL) with a concentration of 50 mg/L (AR18, AY23, BR46) or 10 mg/L (BV10) and pH values of 2–11 were added to the flasks, which were then placed on a multi-station shaker (150 r.p.m.; 30 mm vibration amplitude) for 120 min. Afterwards, samples of the solutions were taken from the flasks to earlier prepared test tubes for analysis of the concentration of dye left in the solution. The post-sorption solutions were also tested for their pH.

### 4.4. Determination of Dye Sorption Kinetics

Portions of the sorbents (10 g d.m. each) were weighed into 1000 mL laboratory beakers. Next, dye solutions (1000 mL) with concentrations of 50/250 mg/L (AR18, AY23, BV10) or 250/1000 mg/L (BR46) and pH values optimal for the sorption process (established as in Section 4.3) were added to the beakers. Then, the beakers were placed on a multi-channel magnetic stirrer (200 r.p.m., 50 × 8 mm Teflon agitator). In specified time intervals (i.e., after 0, 10, 20, 30, 45, 60, 90, 120, 150, 180, 210, 240, and 300 min), samples of the solutions (2 mL) were collected with an automatic pipette and placed into the earlier prepared test tubes.

### 4.5. Determination of the Maximum Sorption Capacity of the Tested Sorbents

Portions of the sorbents (2.5 g d.m. each) were weighed into 1000 mL conical flasks. Next, dye solutions (250 mL) with concentrations of 10–500 mg/L (AR18, AY23, BV10) or 10–1000 mg/L (BR46) with sorption pH values optimal for each dye (established as in Section 4.3) were added to the beakers. Then, the beakers were placed on a multi-station shaker (150 r.p.m.; 30 mm vibration amplitude) for the sorption equilibrium time (determined as in Section 4.4). After the specified time intervals, samples of the solutions were taken from the flasks for analysis of the concentration of dye left in the solution.

#### Comments to Section 4.3, Section 4.4 and Section 4.5

- Each sorbent (NP, LWC, OP, and CC) was tested with respect to 4 dyes (AR18, AY23, BV10, and BR46).
- All dye solutions were prepared based on deionized water.
- All experimental series were performed in three replications.
- Portions of sorbents were weighed on a precise scale with an accuracy of 0.001 g.
- The mixing parameters set on the shaker or multi-channel mixer ensured sorbent mixing throughout the entire solution’s volume.
- Concentrations of dyes left in the solution were determined with the spectrophotometric method, using a UV-VIS spectrophotometer with a cuvette with an optical path length of 10 mm.
- Calibration curves plotted for AR18, AY23, and BR46 dyes enabled determinations in the solution concentration range of 0–50 mg/L, whereas the curve plotted for BV10 allowed for determinations in the solution concentration range of 0–10 mg/L. Solutions with higher concentrations were diluted with deionized water.
- In the case of the analyses conducted in Section 4.3, Section 4.4 and Section 4.5, additional experimental series without dye were performed for each tested sorbent. Aqueous solutions obtained in this way were used to calibrate the spectrophotometer before dye concentration analysis. This procedure enabled the elimination of measurement errors triggered by putative colored contaminants released to the solutions from the waste paper materials.
- The air temperature in the laboratory was kept stable at 25 °C throughout the course of the analyses.

### 4.6. FTIR Analysis, SEM Analysis, and Determination of Specific Surface Area and Porosity of the Tested Sorbents

FTIR analysis of the tested sorbents was carried out using an FT/IR-4700LE spectrophotometer with a diamond crystal single-reflection ATR attachment (JASCO International, Tokyo, Japan). The samples were scanned in a wavelength range of 4000 to 400 cm^−1^. The resolution of each spectrum was 1 cm^−1^. In total, 64 spectra were made for each sample, and the results were averaged. Prior to each measurement, the diamond crystal of the ATR attachment was carefully cleaned with acetone and dried with a paper towel, and then the baseline was corrected.

SEM analysis of the tested materials was performed using a Quanta 200 microscope (FEI, Eindhoven, The Netherlands). The samples were coated with a layer of gold and analyzed under high vacuum conditions at an electron-beam accelerating voltage of 5 KV.

The specific surface areas and porosities of the sorbents were measured using an ASAP 2020 apparatus (Micromeritics, Norcross, GA, USA). Analyses were conducted with the method of low-temperature nitrogen adsorption/desorption. Prior to the analysis, the samples were degassed under vacuum at a temperature of 100 °C for 4 h. The results of the measurements are provided to exactly three significant digits.

### 4.7. Computation Methods

The amount of dye bound on the sorbents was computed using Formula (1):

(1) $$Q_{S}=(C_{0}-C_{S})\times \frac{V}{m}$$

Q_S_—mass of sorbed dye [mg/g];
C_0_—initial concentration of dye [mg/L];
C_S_—concentration of dye after sorption [mg/L];
V—volume of the solution [L];
m—mass of the sorbent [g].

The kinetics of dye sorption onto the tested sorbents was described using pseudo-first-order (2), pseudo-second-order (3), and intraparticular diffusion (4) models:

(2) $$q=q_{e}\times (1-e^{\left(-k_{1}\times t\right)})$$

(3) $$q=\frac{\left(k_{2}\times {q_{e}}^{2}\times t\right)}{\left(1+k_{2}\times q_{e}\times t\right)}$$

(4) $$q=k_{id}\times t^{0.5}$$

q—instantaneous value of sorbed dye [mg/g];
q_e_—the amount of dye sorbed at the equilibrium state [mg/g];
t—time of sorption [min];
k_1_—pseudo-first-order adsorption rate constant [1/min];
k_2_—pseudo-second-order adsorption rate constant [g/(mg × min)];
k_id_—intraparticular diffusion model adsorption rate constant [mg/(g × min^0.5^)].

Experimental data from determinations of the maximum sorption capacity of the NP, LWC, OP, and CC towards AR18, AY23, BV10, and BR46 were described by means of the three most common sorption models: Langmuir 1 isotherm (5), Langmuir 2 isotherm (Langmuir double isotherm) (6), and Freundlich isotherm (7).

(5) $$Q=\frac{\left(Q_{max}\times K_{C}\times C\right)}{\left(1+K_{C}\times C\right)}$$

(6) $$Q=\frac{\left(b_{1}\times K_{1}\times C\right)}{\left(1+K_{1}\times C\right)}+\frac{\left(b_{2}\times K_{2}\times C\right)}{\left(1+K_{2}\times C\right)}$$

(7) $$Q=K\times C^{\frac{1}{n}}$$

Q—mass of sorbed dye [mg/g];
Q_max_—maximum sorption capacity in Langmuir equation [mg/g];
b_1_—maximum sorption capacity of sorbent (type I active sites) [mg/g];
b_2_—maximum sorption capacity of sorbent (type II active sites) [mg/g];
K_C_—constant in Langmuir equation [L/mg];
K_1_,K_2_—constants in Langmuir 2 equation [L/mg];
K—the equilibrium sorption constant in the Freundlich model;
n—Freundlich equilibrium constant;
C—concentration of the dye remaining in the solution [mg/L].

## 5. Conclusions

The waste paper materials investigated in the present study may find application as unconventional sorbents for the removal of dyes from aqueous solutions. They ensure a particularly high effectiveness of BV10 and BR46 sorption. The sorption capabilities of NP, LWC, and CC towards cationic dyes are comparable with those of activated carbons.

The most effective BV10 and BR46 sorbent was revealed to be NP, with sorption capacities of 38.87 mg/g and 90.82 mg/g, respectively. The analyzed sorbents’ effectiveness of cationic dye sorption decreased in the following order: NP > CC > LWC > OP. Presumably, the lignin contents of the sorbents (NP > CC > LWC > OP) was the major contributor to their sorption capability, as it was tantamount to the mentioned order of BV10 and BR46 sorption effectiveness. The positive impact of lignin on the sorbents’ capacity for binding cationic dyes, compared to cellulose, may be due to its higher number of active sites capable of forming hydrogen bonds with the atoms of functional groups in these dyes. The poor sorption potential of OP towards cationic dyes could stem from its lack of lignin and its high content of calcite, which exhibits a very poor capability to bind compounds of an alkaline nature.

The analyzed waste paper materials’ sorption effectiveness of anionic dyes was relatively low. Like in the case of BV10 and BR46, NP proved to be the most effective sorbent of AR18 and AY23. In the case of the anionic dyes, the sorbents performed in a similar way as for the cationic dyes, i.e.: NP > CC > OP > LWC. This finding suggests that lignin also has a positive effect on the sorption effectiveness of AR18 and AY23. This positive effect stems from the fact that—compared to cellulose—lignin may potentially have a higher number of protonated hydroxyls groups, which are important sorption centers for anionic dyes. LWC sorption of AR18 and AY23 was less effective than that by OP, despite it having a higher lignin content. This may be explained by the kaolin coating, typical of LWM, which exhibits a very low capability to bind anionic dyes. This pigment coat found on the sorbent’s surface could, additionally, block access to the functional groups of cellulose and lignin located beneath it.

The substantially poorer capabilities of NP, LWC, OP, and CC to bind to anionic dyes compared to cationic dyes could be due the lack of typically alkaline functional groups (e.g., amine or acetamide groups), which represent the best type of sorption center for anionic dyes.

The effectiveness of dye sorption on the analyzed waste paper sorbents was strongly determined by pH solution. The effectiveness of AR18, AY23, and BV10 sorption were the highest at pH 2, and that of RB46 at pH 6. The high effectiveness of BV sorption at a low pH (pH 2), which is untypical for a cationic dye, is most likely attributed to its carboxyl functional group (-COOH). Its alkaline nature facilitated dye binding in the acidic environment.

The influence of pH on the sorption effectiveness of NP, LWC, OP, and CC for the all analyzed dyes was very alike, suggesting that all the waste paper-based materials share a similar chemical nature.

The analyzed waste paper sorbents modified the pH of the dye solutions during sorption. This was due to the acceptance of protons by the sorbent’s surface at low pH values, or their donation at high pH values, which ultimately led to the ionization of functional groups. Solutions in systems always tend to achieve pH values approximating the pH_PZC_ of the sorbent. The pH_PZC_ values of the analyzed sorbents, determined with the “drift” method, reached pH 7.85 for NP, pH 8.74 for LWC, pH 8.52 for OP, and 8.20 for CC.

The dye sorption equilibrium times recorded for the analyzed waste paper sorbents ranged from 60 to 210 min and depended mainly on the initial dye concentration in the solution, but also on sorbent type. Generally, shorter sorption times were achieved with higher initial dye concentrations, which was presumably due to the greater likelihood of collisions between sorbate molecules and the sorption centers of the sorbents, as well as faster saturation of their active sites. The longer duration of dye sorption on CC compared to NP, LWC or OP could stem from the significantly greater thickness of the CC sheets and its higher grammage (g/m^2^). The greater thickness of the sorbent strongly impaired the dyes’ access to its interior active sites, which substantially elongated the sorption process.

The kinetics of AR18, AY23, BV10, and BR46 sorption on the waste paper sorbents is best described using the pseudo-second-order model, which is typical for the sorption of organic dyes onto biosorbents. The description of experimental data with the intraparticular diffusion model indicates that dye sorption on the tested sorbents proceeded in two main phases that differ in intensity and duration.

The analysis of constants determined from the Langmuir 1 and Langmuir 2 models shows that only one type of a sorption center played a key role in the sorption of anionic dyes onto waste paper sorbents. Most likely, these were the few protonated hydroxyl groups of both cellulose and lignin. In turn, the sorption of cationic dyes onto the analyzed materials was strongly influenced by at least two types of active sites. In the case of BR46, these were presumably the hydrogen atoms of the hydroxyl groups of sorbents forming strong hydrogen bonds with the dye’s nitrogen atoms, as well as the oxygen atoms of the hydroxyl and carbonyl groups of the sorbent forming weak hydrogen bonds with the hydrogen atoms of the functional groups of BR46. In the case of BV10, these could additionally be protonated hydroxyl functional groups reacting electrostatically with the ionized carboxyl group of BV10.

There were no clear correlations between the sorbents’ surface morphology (BET specific surface area, porosity, pore diameter) and their maximum sorption capacities, which may be due to the changes in the structure of the waste paper sorbents triggered by their contact with water.

The analyzed materials had little impact on the “color” of the water (APHA color) they came into contact with (color—a water transparency parameter expressed in Hazen units [mg Pt-Co/L]). The water’s color could be attributed to substances released by the waste paper materials, like pigments, lignin, mineral fillers (calcite, kaolin), glue components (e.g., starch), and also various contaminants in dissolved or suspended forms. The intensity of water coloration caused by NP, LWC, and CC depended on pH and increased in the following order: pH 2 < pH 7 < pH 9 < pH 11. This was linked to the growing lignin concentration in the solution in the respective order. The only material that probably released a colored substance into water was LWC, as indicated by the peak visible at 400–700 nm in the UV-VIS spectrum of this solution. However, in the case of each tested sorbent, the intensity of water coloration was very low. At a sorption material dose of 10 g/L, the maximal color value achieved in each experimental series did not exceed 100 mg Pt-Co/L, which suggests that using waste paper as a sorbent does not pose any severe risk of aquatic environment contamination.

## Figures and Tables

**Table 1:** Results of measurements of low-temperature nitrogen adsorption/desorption (BET).

Tested Sorbent	BET Surface Area [m^2^/g]	Pore Volume [cm^3^/g]	Pore Size (Average) [nm]
NP	0.488 ± 0.0331	0.00212	25.0
LCW	2.380 ± 0.0636	0.02100	18.8
OP	1.280 ± 0.0255	0.00862	15.3
CC	0.789 ± 0.0182	0.00532	19.8

**Table 2:** Coloration intensity of the aqueous solutions after introduction of the analyzed waste paper sorbents (NP, LCW, OP, CC; –10 g/L dose). Color of the solution (color APHA) is expressed in Hazen units (mg Pt-Co/L).

Solution pH	Color of Aqueous Solution after 72 h Contact with Tested Materials; 10 g/L Material Dose. Color APHA—[mg Pt-Co/L]				Tap Water (at Our Laboratory)
	NP	LWC	OP	CC	
2	6.95	11.56	8.16	24.05	3.13 [mg Pt-Co/L]
7	12.32	26.71	12.60	34.47	
9	20.34	30.87	14.52	41.22	
11	27.94	82.58	14.63	46.87	

**Table 3:** Kinetic parameters of dye sorption onto NP, LCW, OP, CC, determined using the pseudo-first-order and pseudo-second-order models.

Sorbent	Dye	Dye Conc. [mg/L]	Pseudo-First-Order Model			Pseudo-Second-Order Model			Exp. Data	Equil. Time
			k_1_	q_e, cal._	R^2^	k_2_	q_e, cal_.	R^2^	q_e, exp._	[min]
		[mg/L]	[1/min]	[mg/g]	-	[g/mg × min]	[mg/g]	-	[mg/g]	[min]
NP	AR18	50	0.0939	2.16	0.9912	0.1053	2.29	0.9974	2.20	90
		250	0.1947	5.25	0.9955	0.0759	5.38	0.9995	5.29	60
	AY23	50	0.1028	2.16	0.9920	0.0861	2.28	0.9973	2.19	90
		250	0.1603	5.00	0.9939	0.0775	5.16	0.9993	5.04	60
	BV10	50	0.0630	4.60	0.9886	0.0200	4.98	0.9982	4.69	120
		250	0.0741	19.46	0.9920	0.0060	20.85	0.9968	19.71	90
	BR46	250	0.0999	24.04	0.9955	0.0075	25.33	0.9967	24.29	90
		1000	0.1815	75.03	0.9939	0.0064	77.06	0.9990	75.84	60
LWC	AR18	50	0.0856	1.09	0.9889	0.2306	1.16	0.9975	1.11	90
		250	0.1895	2.09	0.9915	0.1333	2.15	0.9993	2.09	60
	AY23	50	0.0913	1.06	0.9932	0.1460	1.13	0.9959	1.07	90
		250	0.1576	2.02	0.9919	0.0988	2.09	0.9988	2.04	60
	BV10	50	0.0722	3.32	0.9948	0.0337	3.56	0.9960	3.67	120
		250	0.0870	11.51	0.9906	0.0127	12.22	0.9980	11.68	90
	BR46	250	0.0935	22.77	0.9937	0.0071	27.09	0.9967	23.09	90
		1000	0.1790	53.97	0.9933	0.0067	55.46	0.9988	54.56	60
OP	AR18	50	0.0924	1.30	0.9944	0.1223	1.37	0.9953	1.30	90
		250	0.1387	2.28	0.9863	0.1209	2.37	0.9987	2.29	60
	AY23	50	0.0951	1.18	0.9920	0.1989	1.25	0.9967	1.20	90
		250	0.1725	2.28	0.9949	0.1360	2.35	0.9990	2.30	60
	BV10	50	0.0606	1.31	0.9897	0.0657	1.43	0.9967	1.32	120
		250	0.0906	3.39	0.9959	0.0458	3.59	0.9964	3.44	90
	BR46	250	0.0808	8.30	0.9908	0.0258	8.85	0.9971	8.42	90
		1000	0.1631	15.13	0.9887	0.0155	15.63	0.9978	15.37	60
CC	AR18	50	0.0558	2.13	0.9845	0.0361	2.33	0.9992	2.19	150
		250	0.0880	4.70	0.9926	0.0272	5.13	0.9946	4.81	120
	AY23	50	0.0890	2.02	0.9884	0.0730	2.15	0.9981	2.08	150
		250	0.1261	4.66	0.9959	0.0562	4.85	0.9967	4.71	120
	BV10	50	0.0257	4.34	0.9873	0.0059	5.11	0.9939	4.38	210
		250	0.0251	18.26	0.9879	0.0014	21.57	0.9920	18.19	150
	BR46	250	0.0238	22.50	0.9888	0.0010	26.83	0.9949	22.81	210
		1000	0.0280	61.28	0.9859	0.0005	71.26	0.9928	61.23	150

**Table 4:** Dye diffusion rate constants determined using the intraparticular diffusion model.

Sorbent	Dye	Dye Conc.	Phase I			Phase II		
			k_d1_	Durat.	R^2^	k_d2_	Durat.	R^2^
		[mg/L]	[mg/(g × min^0.5^)]	[min]	-	[mg/(g × min^0.5^)]	[min]	-
NP	AR18	50	0.4126	20	0.9953	0.0758	70	0.9604
		250	1.4461	10	0.9999	0.1514	50	0.9444
	AY23	50	0.4244	20	0.9931	0.0667	70	0.9623
		250	1.3008	10	0.9999	0.1965	50	0.9412
	BV10	50	0.7053	30	0.9944	0.1664	90	0.9656
		250	3.4016	20	0.9992	0.9134	70	0.9650
	BR46	250	4.6736	20	0.9954	0.7335	70	0.9316
		1000	20.3320	10	0.9999	2.5411	50	0.9646
LWC	AR18	50	0.2039	20	0.9989	0.0434	70	0.9825
		250	0.5737	10	0.9999	0.0571	50	0.9676
	AY23	50	0.2042	20	0.9998	0.0343	70	0.9758
		250	0.5228	10	0.9999	0.0802	50	0.9448
	BV10	50	0.5473	30	0.9985	0.0878	90	0.9386
		250	2.1564	20	0.9985	0.4622	70	0.9623
	BR46	250	4.3954	20	0.9991	0.7680	70	0.9672
		1000	14.6150	10	0.9999	1.8556	50	0.9537
OP	AR18	50	0.2487	20	0.9994	0.0398	70	0.9661
		250	0.5755	10	0.9999	0.1003	50	0.9796
	AY23	50	0.2265	20	0.9972	0.0357	70	0.9745
		250	0.6072	10	0.9999	0.0803	50	0.9269
	BV10	50	0.2021	30	0.9981	0.0460	90	0.9561
		250	0.6404	20	0.9991	0.1121	70	0.9416
	BR46	250	1.4912	20	0.9971	0.3604	70	0.9568
		1000	4.0086	10	0.9999	0.6008	50	0.9920
CC	AR18	50	0.3127	30	0.9967	0.0718	120	0.9570
		250	0.8837	20	0.9999	0.1237	100	0.9082
	AY23	50	0.3436	30	0.9839	0.0372	120	0.9669
		250	0.9565	20	0.9994	0.0693	100	0.9011
	BV10	50	0.3847	120	0.9929	0.0502	90	0.9008
		250	1.7042	90	0.9986	0.7042	60	0.9946
	BR46	250	1.9793	120	0.9969	0.3457	90	0.9050
		1000	5.8362	90	0.9957	2.1081	60	0.9518

**Table 5:** Constants determined from Langmuir 1, Langmuir 2, and Freundlich models.

Sorbent	Dye	Langmuir 1 Model			Langmuir 2 Model						Freundlich Model		
		Q_max_	K_c_	R^2^	Q_max_	b_1_	K_1_	b_2_	K_2_	R^2^	k	n	R^2^
		[mg/g]	[L/mg]	-	[mg/g]	[mg/g]	[L/mg]	[mg/g]	[L/mg]	-	-	-	-
NP	AR18	7.77	0.014	0.9916	7.77	3.86	0.014	3.91	0.014	0.9916	0.367	0.529	0.9693
	AY23	7.20	0.014	0.9851	7.20	3.60	0.014	3.60	0.014	0.9851	0.355	0.523	0.9402
	BV10	33.53	0.036	0.9759	38.87	6.90	0.804	31.97	0.015	0.9957	4.417	0.377	0.9879
	BR46	78.01	0.054	0.9888	90.82	38.88	0.158	51.94	0.012	0.9991	12.532	0.344	0.9590
LWC	AR18	2.64	0.017	0.9945	2.64	1.52	0.017	1.12	0.017	0.9945	0.194	0.451	0.9602
	AY23	2.54	0.018	0.9908	2.54	1.27	0.018	1.27	0.018	0.9908	0.191	0.447	0.9552
	BV10	21.52	0.010	0.9951	23.22	1.90	0.130	21.32	0.007	0.9975	1.090	0.482	0.9823
	BR46	55.59	0.034	0.9884	63.28	26.72	0.110	36.56	0.008	0.9994	8.376	0.321	0.9581
OP	AR18	2.89	0.019	0.9943	2.89	1.43	0.019	1.46	0.019	0.9943	0.229	0.441	0.9615
	AY23	3.01	0.016	0.9902	3.01	1.39	0.016	1.62	0.016	0.9902	0.204	0.464	0.9544
	BV10	5.89	0.007	0.9889	7.28	6.03	0.043	1.25	0.003	0.9959	0.239	0.490	0.9872
	BR46	18.41	0.006	0.9926	19.61	2.35	0.044	17.26	0.004	0.9947	0.911	0.430	0.9719
CC	AR18	6.62	0.016	0.9918	6.62	3.31	0.016	3.31	0.016	0.9918	0.375	0.501	0.9549
	AY23	6.62	0.013	0.9901	6.62	3.24	0.013	3.38	0.013	0.9901	0.299	0.534	0.9642
	BV10	24.66	0.052	0.9787	28.87	11.20	0.220	17.67	0.011	0.9929	4.262	0.326	0.9651
	BR46	67.36	0.020	0.9896	71.27	7.49	0.607	63.78	0.013	0.9965	7.190	0.372	0.9736

**Table 6:** Sorption capacities of various sorbents and activated carbons towards anionic dyes AR18 and AY23.

Dye	Sorbent	Sorption Capacity [mg/g]	pH of Sorption	Time of Sorption [min]	Source
AR18	Activated carbon WG-12	100.00	–	–	[101]
	Biochar from *Phragmites australis*	95.22	2	720	[102]
	Activated carbon from curry tree seeds	53.19	-	120	[103]
	Rapeseed husk	49.4	2	150	[18]
	Granular activated carbon	45.45	9	120	[104]
	Activated carbon from carrot waste	41.00	3	80	[85]
	Chitosan flakes	39.90	4	180	[86]
	Activated carbon from peach stone	34.24	3	480	[105]
	Activated carbon from poplar wood	30.30	5	120	[87]
	Carboxymethyl cellulose	29.70	6	120	[86]
	Acidic treated pumice	29.70	3.5	180	[106]
	Granular ferric hydroxide	29.13	5	85	[107]
	Reduced graphenoxide/attapulgite	26.59	2	30	[108]
	Magnetite nanoparticles	16.25	3	120	[109]
	*Sargassum glaucescens* biomass	15.0	6	60	[84]
	Compost	13.51	7	1440	[110]
	Nano-pumice	12.84	4.5	90	[107]
	Zeolite	11.10	7	150	[111]
	Activated charcoal almond shell	10.75	2	60	[112]
	Powdered yeast	10.16	3	120	[113]
	Agar	10.16	6	120	[86]
	Newsprint paper (used)—NP	7.77	2	90	This work
	Red mud	7.14	3	75	[114]
	Corrugated cardboard (used)—CC	6.62	2	150	This work
	Office paper (used)—OP	2.89	2	90	This work
	Lightweight coated paper (used)—LWC	2.64	2	90	This work
	Sunflower seed hulls (SSHs)	1.80	3	90	[69]
	Coconut Shells	0.66	2	45	[81]
AY23	Activated carbon of *Lantana Camara*	58.82	2	30	[73]
	Commercial activated carbon	56.50	8	120	[88]
	Amberlite IRA-900	49.88	4.5	20	[115]
	Rapeseed husks	41.52	2	150	[18]
	Chitin	30.50	3	240	[116]
	Deoiled soya	24.60	2	-	[117]
	Chitin flakes	24.20	2	120	[72]
	Activated carbon-based cola nut shells	21.59	2	10	[74]
	Activated carbon from *Cassava sievate*	20.83	2	90	[75]
	Bottom ash	12.60	2	-	[117]
	Newsprint paper (used)—NP	7.20	2	90	This work
	Corrugated cardboard (used)—CC	6.62	2	150	This work
	Sawdust	4.71	3	70	[70]
	Cotton fibers	3.58	3	240	[71]
	Office paper (used)—OP	3.01	2	90	This work
	Lightweight coated paper (used)—LWC	2.54	2	90	This work
	Sunflower seed hulls (SSHs)	2.30	3	90	[69]
	Activated carbon from coconut shell	2.30	1.7	60	[118]
	Coconut shells	0.53	2	45	[81]
	Organobentonite/alginate hydrogel	0.50	4.5	-	[119]

**Table 7:** Sorption capacities of various sorbents and activated carbons towards cationic dyes BV10 and BR46.

Dye	Sorbent	Sorption Capacity [mg/g]	pH of Sorption	Time of Sorption [min]	Source
BV10	Newsprint paper (used)—NP	38.87	2	120	This work
	Activated carbon (palm shell-based)	30.00	3	-	[120]
	Activated carbon from jute fiber	28.00	8	220	[121]
	Corrugated cardboard (used)—CC	24.66	2	210	This work
	Lightweight coated paper (used)—LWC	21.52	2	120	This work
	Rapeseed husks	20.90	3	180	[68]
	Banana peels	20.60	7	1440	[122]
	Municipal solid waste compost	19.30	3	1440	[123]
	Cedar cones	17.20	5	480	[95]
	Coconut fiber	14.90	9.2	90	[90]
	Sugar cane fiber	10.40	-	-	[124]
	Molts of mealworm	6.44	3	210	[79]
	Office paper (used)—OP	5.89	2	120	This work
	Lemon peels	5.70	3	240	[80]
	Unmodified activated sludge	4.60	6.5	120	[125]
	Cedar cones	4.60	0	720	[95]
	Grapefruit peels	4.60	3	240	[80]
	*Calotropis procera* leaf biomass	4.10	-	60	[89]
	Champignon biomass	4.00	2	210	[76]
	Mango leaves (powder)	3.30	-	50	[126]
	Chitin from the molts of mealworms	3.22	6	120	[91]
	Orange peels	3.20	4	240	[80]
	Chitosan (non-cross-linked granules)	2.94	6	1440	[127]
	Coal-fired coconut fiber	2.60	6.5	150	[128]
	Powdered coffee	2.50	2	180	[77]
	Fly ash washed with NaOH	2.50	6.2	4320	[129]
BR46	Activated carbon Chemviron GW	106.00	7.4	120	[130]
	Newsprint paper (used)—NP	90.82	6	90	This work
	Corrugated cardboard (used)—CC	71.27	6	210	This work
	Activated carbon from biomass	65.70	7	90	[131]
	Lightweight coated paper (used)—LWC	63.28	6	90	This work
	Rapeseed hulls	59.10	6	180	[18]
	TiO_2_ nanopart. loaded on activ. carbon	58.61	5.5	4	[132]
	Spent green tea leaves	58.00	6	240	[35]
	Lemon peels	54.00	6	240	[80]
	Molts of mealworm (MM)	50.90	6	150	[79]
	Coconut shells	49.40	6	120	[81]
	Activated carbon ROW 08	45.00	8	60	[133]
	*Paulownia tomentosa* tree leaves	43.10	8	72	[134]
	Gypsum	39.17	10	60	[135]
	Single-walled carbon nanotubes	38.35	9	100	[136]
	Biochar from *Chrysanthemum morifolium* straw	32.30	10	60	[137]
	Exoskeletons of mealworm (ME)	31.50	6	180	[79]
	Nut sawdust	30.10	7	-	[138]
	Activated carbon	26.41	5	4	[132]
	Natural sugarcane stalks powder	20.96	7.2	60	[92]
	Bone meal	24.60	6	90	[93]
	Office paper (used)—OP	19.61	6	90	This work
	Beech sawdust	19.24	-	150	[94]
	Wood sawdust	19.20	-	120	[94]
	Kaolin	12.80	6.6	60	[99]
	Hen feathers	4.06	5	210	[78]

**Table 8:** Parameters of the analyzed sorbents acc. to the literature data [38,41,139,140,141,142,143,144].

Waste Paper Type	Grammage [g/m^2^]	Percentage Content [%]			
		Cellulose	Hemicellulose	Lignin	Others (Fillers, Glues, Inks)
NP	50	38.0–55.0	18.0–40.0	18.0–30.0	1.0–6.0
LWC	60	28.0–46.0	17.0–32.0	6.0–17.2	20.0–30.0
OP	80	60.0–78.6	4.7–14.2	0.9–1.0	12.3–30.0
CC	350	58.0–60.0	14.0–15.0	13.6–15.4	7.7–20.0

**Table 9:** Characteristics of dyes used in this study.

Dye Name	Acid Red 18 (AR18)	Acid Yellow 23 (AY23)	Basic Violet 10 (BV10)	Basic Red 46 (BR46)
Structural formula	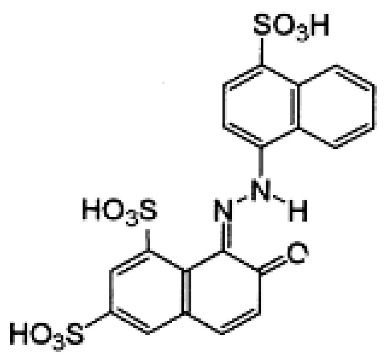	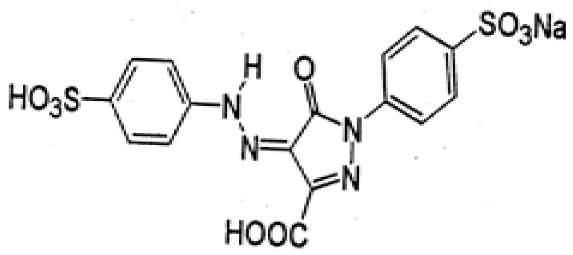	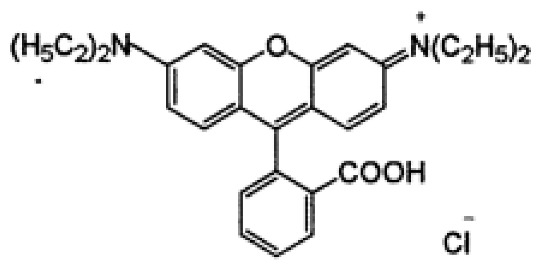	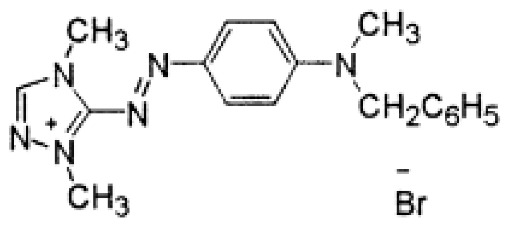
Chemical formula	C_20_H_11_N_2_Na_3_O_10_S_3_	C_16_H_9_N_4_Na_3_O_9_S_2_	C_28_H_31_ClN_2_O_3_	C_18_H_21_BrN_6_
Molecular weight	604.5 g/mol	534.4 g/mol	479.0 g/mol	321.4 g/mol
Dye class	single azo dye	single azo dye	xanthene dye	single azo dye
Dye type	anionic (acidic)	anionic (acidic)	cationic (basic)	cationic (basic)
UV-VIS spectrum of dye	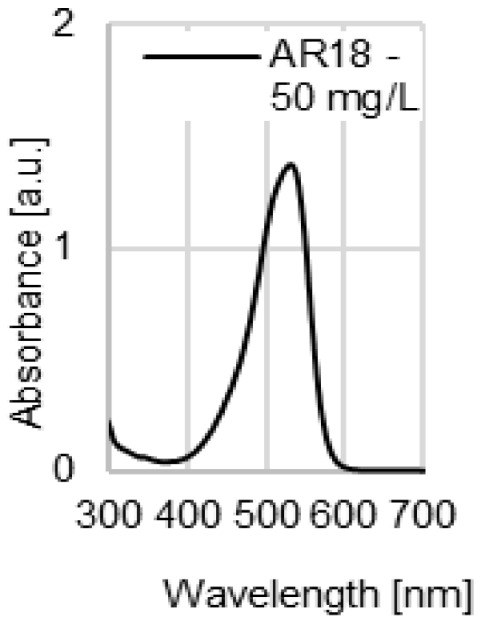	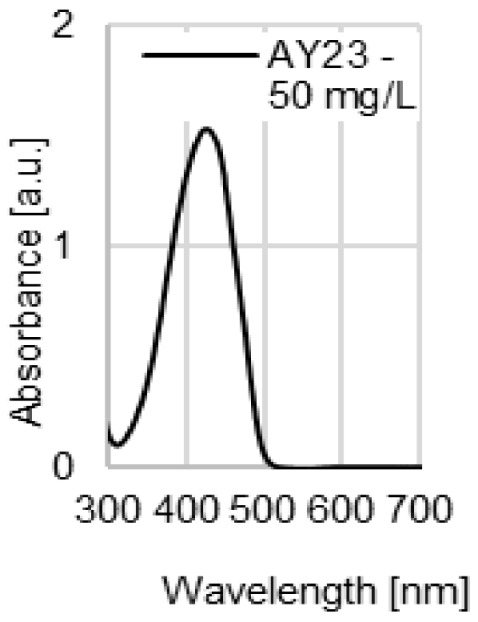	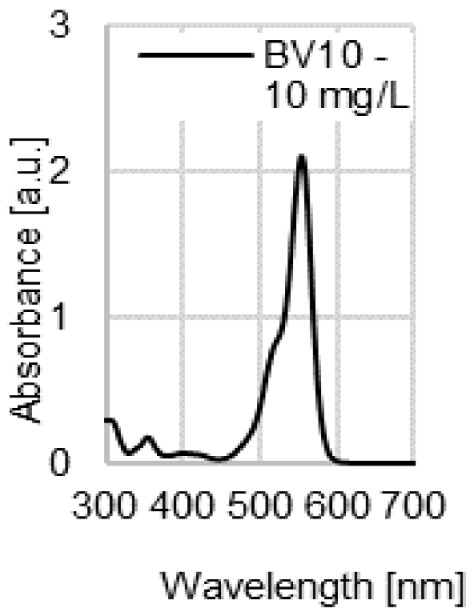	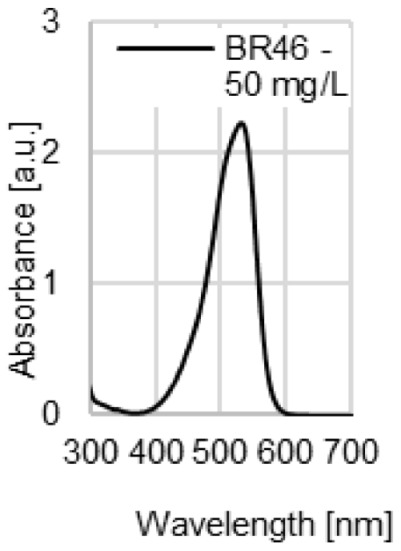
λ_max_	509 nm	428 nm	554 nm	530 nm
Uses	Dyeing wool, silk, polyamide fiber	Dyeing wool, silk, polyamide fiber	Dyeing textiles, paper, leather	Dyeing leather, paper, wool, and acrylic fibers
Other trade names	Acid Brilliant Red 3R, Acid Scarlet 3R	Tartrazine, Acid Tartrazine	Rhodamine B, Basic Red RB	Anilan Red GRL, Basic Red X-GRL

## Data Availability

The data presented in this study are available on request from the corresponding author.

## Supplementary Materials

The following supporting information can be downloaded at: https://www.mdpi.com/article/10.3390/molecules29122809/s1, Figure S1. Sorption kinetics of anionic dyes (a) AR18—50 mg/L, (b) AR18—250 mg/L, (c) AY23—50 mg/L, and (d) AY23—250 mg/L onto: NP, LCW, OP, CC (10 g/L dose) (average + range). Pseudo-first-order model and pseudo-second-order model; Figure S2. Sorption kinetics of basic dyes (a) BV10—50 mg/L, (b) BV10—250 mg/L, (c) BR46—250 mg/L, and (d) BR46—1000 mg/L onto NP, LCW, OP, and CC (10 g/L dose) (average + range). Pseudo-first-order model and pseudo-second-order model; Figure S3. Intraparticular diffusion model of the sorption of (a) AR18—50 mg/L, (b) AR18—250 mg/L, (c) AY23—50 mg/L, and (d) AY23—250 mg/L onto NP, LCW, OP, and CC (10 g/L dose); Figure S4. Intraparticular diffusion model of the sorption of (a) BV10—50 mg/L, (b) BV10—250 mg/L, (c) BR46—250 mg/L, and (d) BR46—1000 mg/L onto NP, LCW, OP, and CC (10 g/L dose).
