# Supplementary material for: The Use of Various Types of Waste Paper for the Removal of Anionic and Cationic Dyes from Aqueous Solutions
Source: Molecules. 2024 Jun 12;29(12):2809. doi: 10.3390/molecules29122809 (PMC11206315; doi:10.3390/molecules29122809)
Supplement: Supplementary file 1 [file molecules-29-02809-s001.zip › molecules-3012857-supplementary.pdf]

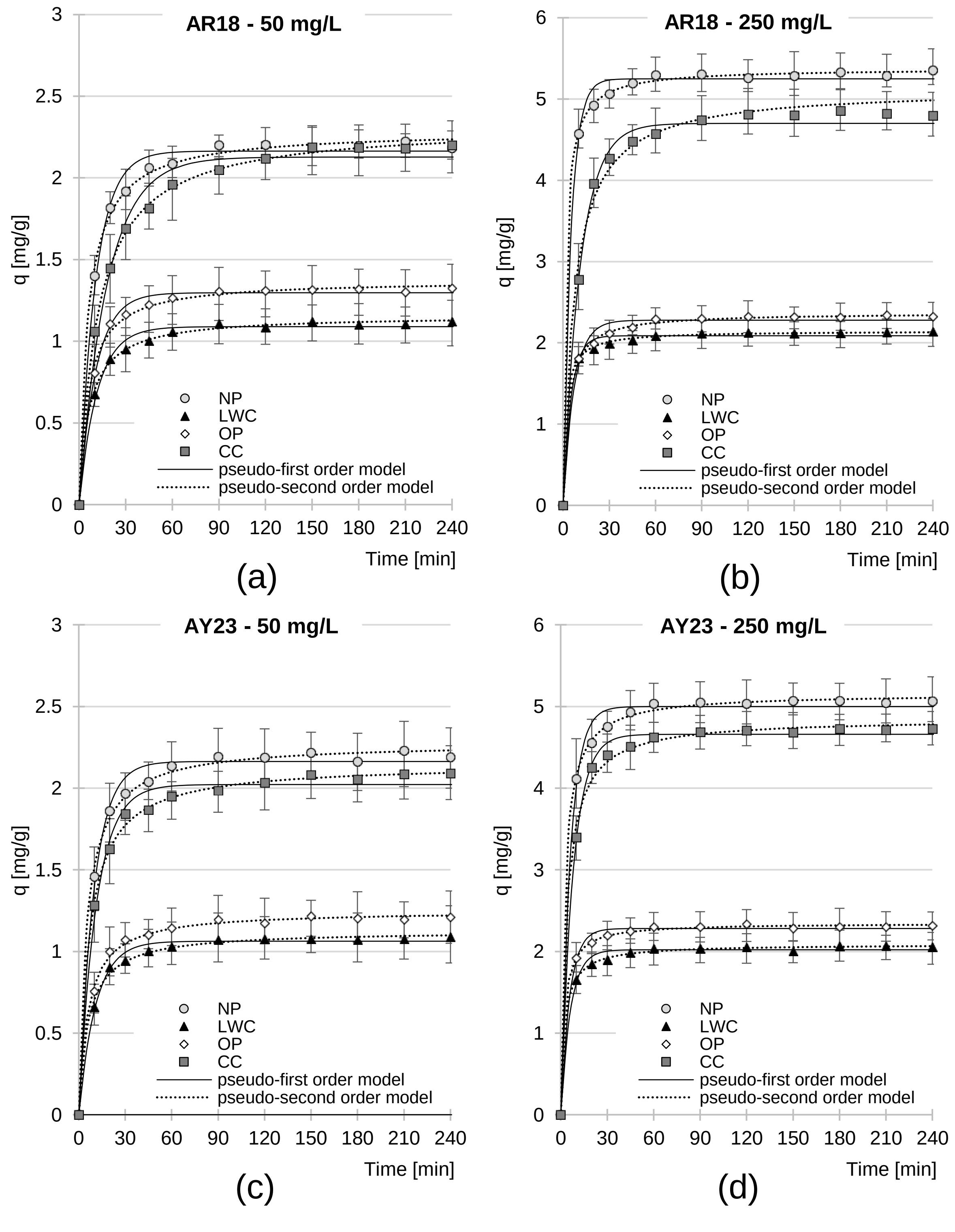

Figure S1. Sorption kinetics of anionic dyes (a) AR18 – 50 mg/L, (b) AR18 – 250 mg/L, (c) AY23 – 50 mg/L, and (d) AY23 – 250 mg/L onto: NP, LCW, OP, CC (10 g/L dose) (average + range). Pseudo-first-order model and pseudo-second-order model

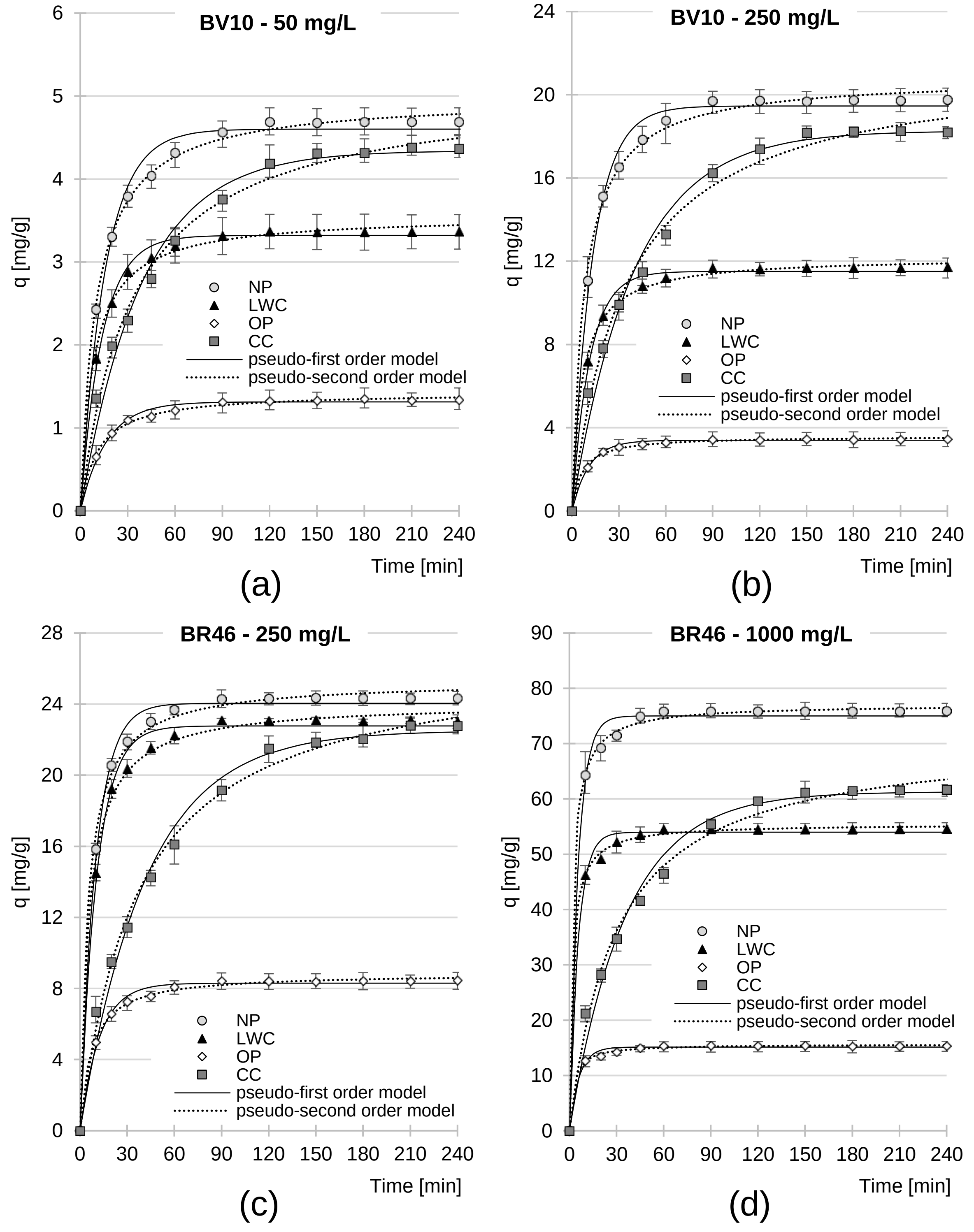

Figure S2. Sorption kinetics of basic dyes (a) BV10 – 50 mg/L, (b) BV10 – 250 mg/L, (c) BR46 – 250 mg/L, and (d) BR46 – 1000 mg/L onto: NP, LCW, OP, CC (10 g/L dose) (average + range). Pseudo-first-order model and pseudo-second-order model

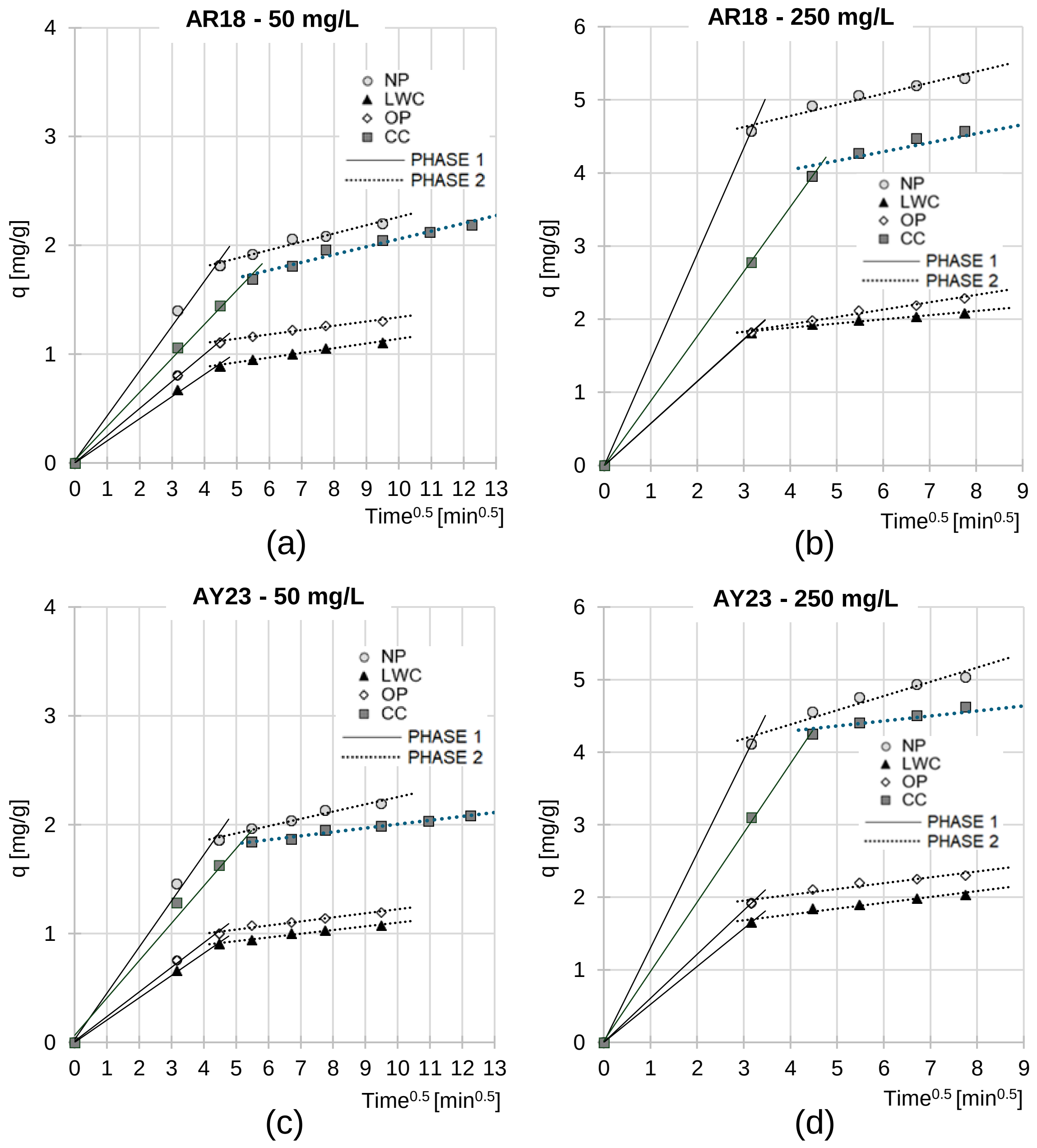

Figure S3. Intraparticular diffusion model of the sorption of: (a) AR18 – 50 mg/L, (b) AR18 – 250 mg/L, (c) AY23 – 50 mg/L, and (d) AY23 – 250 mg/L onto: NP, LCW, OP, and CC (10 g/L dose)

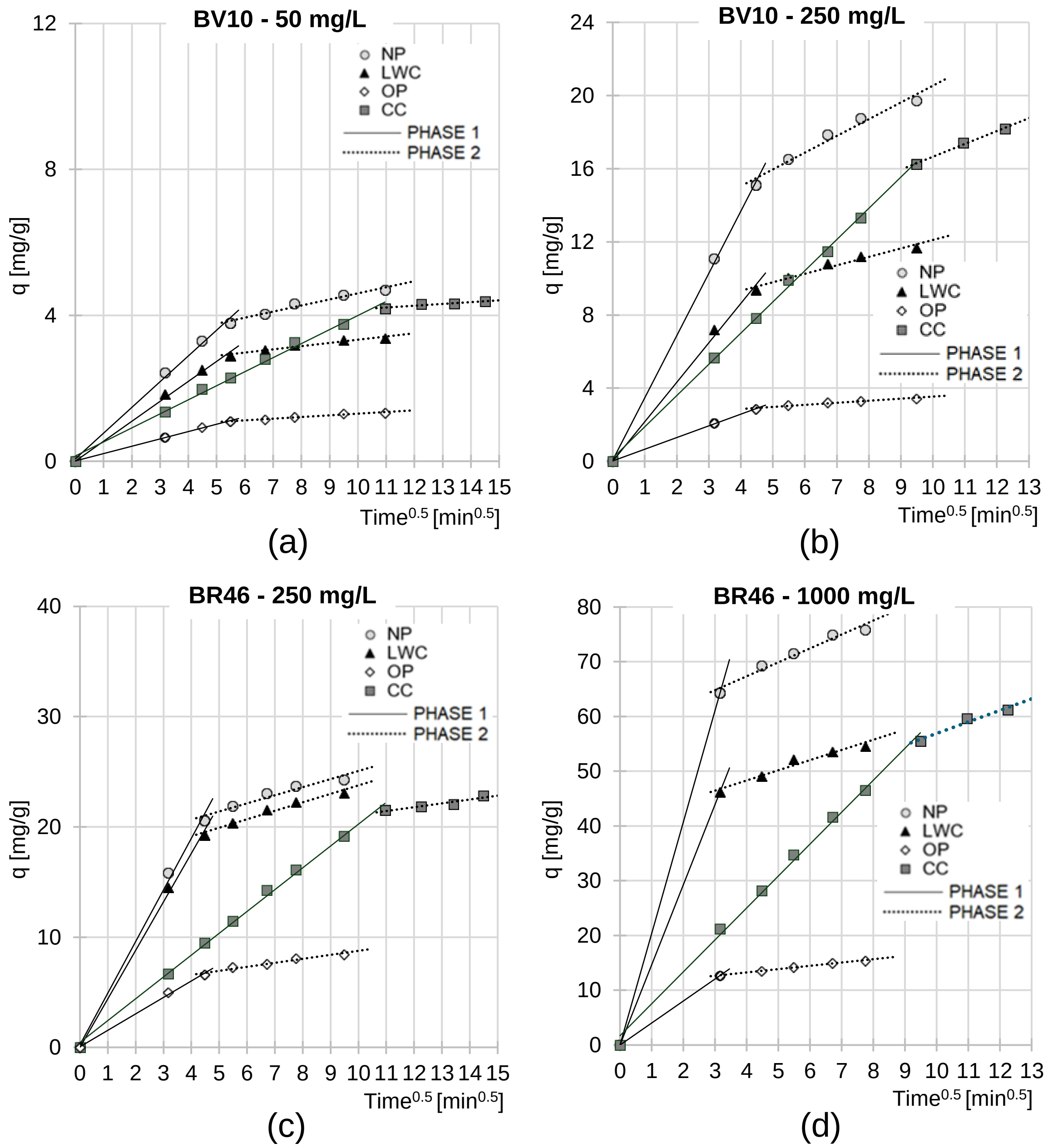

Figure S4. Intraparticular diffusion model of the sorption of: (a) BV10 – 50 mg/L, (b) BV10 – 250 mg/L, (c) BR46 – 250 mg/L, and (d) BR46 – 1000 mg/L onto: NP, LCW, OP, and CC (10 g/L dose)
